# Constraining size-dependence of vegetation respiration rates

**DOI:** 10.1038/s41598-020-61239-0

**Published:** 2020-03-09

**Authors:** Akihiko Ito

**Affiliations:** 1 National Institute for Environmental Studies, 16-2 Onogawa, Tsukuba, 305-8506 Japan; 2 Japan Agency for Marine-Earth Science and Technology, 3173-25 Showa-machi, Kanazawa-ku, Yokohama, 236-0001 Japan

**Keywords:** Ecosystem ecology, Carbon cycle, Ecological modelling

## Abstract

Plant autotrophic respiration is responsible for the atmospheric release of about half of all photosynthetically fixed carbon and responds to climate change in a manner different from photosynthesis. The plant mass-specific respiration rate (*r*_A_), a key parameter of the carbon cycle, has not been sufficiently constrained by observations at ecosystem or broader scales. In this study, a meta-analysis revealed a global relationship with vegetation biomass that explains 67–77% of the variance of *r*_A_ across plant ages and biomes. *r*_A_ decreased with increasing vegetation biomass such that annual *r*_A_ was two orders of magnitude larger in fens and deserts than in mature forests. This relationship can be closely approximated by a power-law equation with a universal exponent and yields an estimated global autotrophic respiration rate of 64 ± 12 Pg C yr^−1^. This finding, which is phenomenologically and theoretically consistent with metabolic scaling and plant demography, provides a way to constrain the carbon-cycle components of Earth system models.

## Introduction

The amount of carbon dioxide (CO_2_) assimilated by photosynthesis is nearly equaled by the amount released back into the atmosphere by ecosystem respiration (defined as the sum of autotrophic plant respiration and heterotrophic microbial and animal respiration). Approximately half of the assimilated CO_2_ is released by autotrophic (mostly dark) respiration (*R*_A_), which varies with biotic and abiotic factors^1,2^. Autotrophic respiration, which involves complicated metabolic pathways, is a key determinant of carbon production, use efficiency, turnover, and ecosystem net carbon balance^3,4^. Although few observational and modeling studies have quantified *R*_A_ at the global scale, many studies have estimated gross and net primary production (GPP and NPP, typically in Mg C ha^−1^ yr^−1^ at the ecosystem scale) and soil respiration (ground surface efflux of CO_2_ from plant roots and microbes)^5^. Studies of plant carbon-use efficiency (i.e., = NPP/GPP, or 1 − *R*_A_/GPP; empirically 0.22–0.79) have indicated that *R*_A_ is not a constant fraction of GPP^3,6^. Most terrestrial carbon cycle and vegetation models estimate *R*_A_ in a more simplified form than photosynthesis with respect to both biochemistry and empirical parameterization^7^. Specifically, most of these models parameterize *R*_A_ on the basis of the growth–maintenance respiration paradigm^8,9^ (Supplementary Fig. S1). This scheme is phenomenological and practical, and it allows analysis of how respiration is regulated by cost-based components. However, its key coefficients (mass-specific plant organ respiration rates and environmental responsiveness) are poorly constrained by observations and are arbitrarily calibrated, leading to large carbon cycle uncertainties in Earth system models^10,11^. At present, many land models include a vegetation dynamic in which plant age and mass classes and competitive dynamics between then are explicitly simulated. Therefore, there is a clear need to devise a practical constraint for the behavior of these large-scale models.

### Ecological theories and hypotheses

Here, it is hypothesized that the total plant autotrophic respiration rate (i.e.,including growth and maintenance, from roots to leaves) is size-dependent and should be based on biological constraints. Intuitively, large individuals, which tend to have lower specific surface areas, more inactive organs (woody stems and coarse roots), lower nitrogen concentrations, and slower growth rates (Supplementary Fig. S1), may be expected to have lower respiration rates. A more theoretical and quantitative interpretation is proposed here.

First, the accumulation of organ- and individual-scale measurements of plant gas fluxes and mass balances has led to a metabolic scaling theory of plant respiration^12–14^. These studies have demonstrated a power-law relationship between individual weight (*W*_I_, kg C per individual) and respiration rate (*r*_I_, g C per individual yr^−1^) across a wide range of plant sizes:

1 $${r}_{I}=a\cdot {W}_{I}^{\alpha },$$

where *a* is a coefficient and *α* is the scaling exponent. Despite the increasing availability of global vegetation data (e.g., forest density^15^) thanks to satellite observations and dataset compilation, predicting large-scale (e.g. regional) *R*_A_ from this relationship alone is highly challenging.

Meanwhile, at the population level, competition and self-thinning account for a negative relationship between plant density (*N*, individuals ha^−1^) and mean individual weight (*W*_I_) according to the following power law^16^:

2 $${W}_{I}=b\cdot {N}^{\beta },$$

where *b* is a coefficient and the exponent *β* reflects plant demographic effects. This power-law relationship was first established in studies of ideal monocultures, and the mechanisms underlying the density effect such as resource competition have been investigated^16,17^. Moreover, the relationship has been critically evaluated through studies on real-world vegetation and has now become a recognized ecological principle^17^. When combined with Eq. (1), the relationship between vegetation biomass (*W*_V_ = *N* · *W*_I_, kg C ha^−1^ or Mg C ha^−1^) and mass-specific respiration rate (*r*_A_ = *R*_A_ / *W*_V_, g C kg C^−1^ yr^−1^) can be expressed by the following power-law:

3 $${r}_{{\rm{A}}}=c\cdot {{W}_{{\rm{V}}}}^{\frac{\beta \cdot (\alpha -1)}{(1+\beta )}},$$

where *c* is a coefficient. See Supplementary Information for a detailed derivation of Eq. (3).

## Results and Discussion

### Meta-analysis of scaling relationship

**Figure 1:**
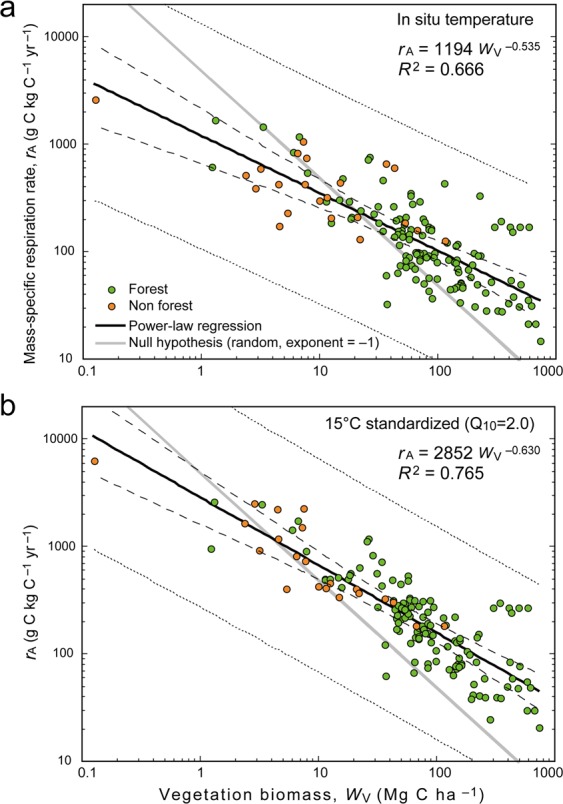
Mass-specific respiration rate (*r*_A_) versus biomass carbon stock (*W*_V_). Comparison of *r*_A_ values (**a**) measured at the *in situ* temperature of the measurement site, and (**b**) standardized to 15 °C. Thick lines show power-law regressions (*n* = 144, *P* < 0.001 for both plots). Regression equations and correlation coefficients are shown in each panel. Dashed lines indicate 95% confidence intervals, and dotted lines show 95% prediction intervals. Gray lines show the slope (i.e., −1.0) of the null model, which assumes that vegetation respiration rate is independent of biomass (under this assumption, any significant trend would merely be due to autocorrelation). *r*_A_ was standardized to 15 °C by using an exponential temperature dependence curve with Q_10_ = 2.0.

**Figure 2:**
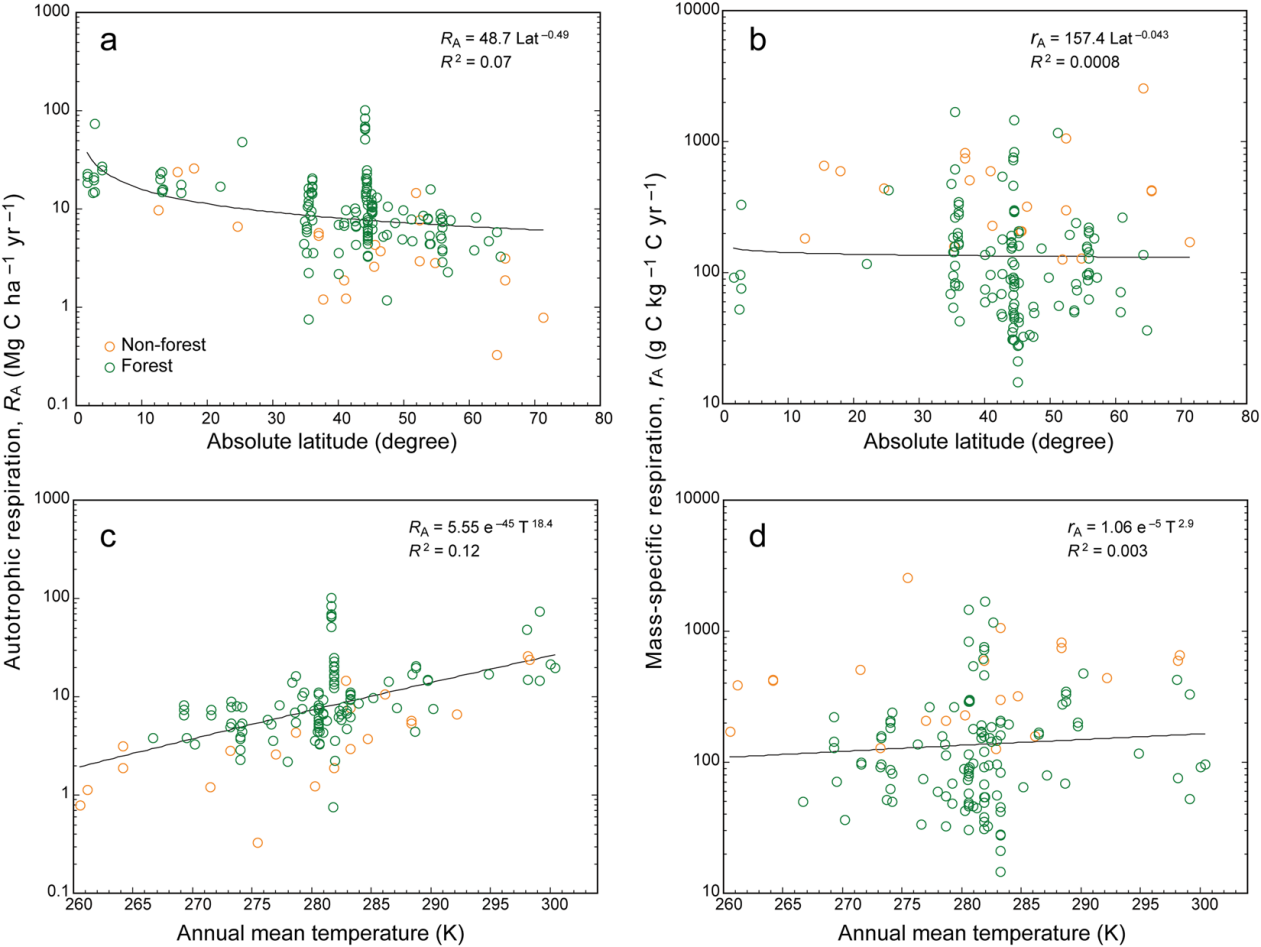
Vegetation respiration rate relationships. Changes in vegetation respiration rate (*R*_A_) and mass-specific respiration rate (*r*_A_) with (**a, b**) latitude and (**c, d**) annual mean temperature. Regression lines, equations, and coefficients of determination (*R*^2^) for the power-law equation are shown in each panel.

To determine the scaling relationship at larger spatial scales, I conducted a meta-analysis of vegetation biomass and *R*_A_ at the ecosystem scale (typically 10^2^–10^6^ m^2^; see Methods for details). I used observational datasets from the literature from a variety of ecosystems ranging from infertile fens and deserts to mature tropical forests (148 records from 73 studies; Supplementary Table S1; Supplementary Fig. S2). The studies used a range of observational methods, including chamber measurements and biometric mass balances, which are each subject to certain errors and biases. Mass-specific respiration rates, *r*_A_, ranged from 14.6 g C kg^−1^ C yr^−1^ in a mature temperate conifer forest to 2588 g C kg^−1^ C yr^−1^ in a boreal peatland. Overall, *r*_A_ was closely correlated with vegetation biomass by a power-law equation (Fig. 1) at both *in situ* and standardized temperatures:

4 $${r}_{{\rm{A}}}=c{\prime} \cdot {W}_{{\rm{V}}}^{\gamma },$$

where *c*′ is a coefficient. *γ* corresponds to the exponent in Eq. (3) and was estimated as −0.535 (95% confidence interval, −0.465 to −0.605) at *in situ* temperatures and −0.630 (−0.565 to −0.694) at 15 °C. This relationship covers a wide range of vegetation types (e.g., forests and non-forests), ages, and densities. The data deviate from the log–log relationship by up to a few orders of magnitude, and the coefficient of determination (*R*^2^) is lower than that for individual-scale studies on metabolic scaling (about 0.9^13,14^). Nevertheless, Eq. (4) explains a remarkable 67–77% of the variance in vegetation *r*_A_, a much higher percentage than explained by latitude and temperature (Fig. 2). As discussed later, data obtained from multiple nearby sites with different disturbance histories (i.e., at at different points in a chronosequence) follow this scaling relationship. Also, the scaling exponent differed substantially from −1 (the exponent of the null model, where *R*_A_ is independent of *W*_V_; see gray lines in Fig. 1), which implies that the relationship is biologically meaningful and does not merely reflect autocorrelation.

The derived relationship is consistent with biological constraints. Previously proposed values of *α* are 2/3 (surface-area to mass scaling), 3/4 (Kleiber’s law, mass–vascular branching), and 1.0 (isometric scaling)^12,13^. Typical values of the demographic coefficient *β* have been posited to be −1.605 (Reineke’s rule), −3/2 (Yoda’s rule), and −4/3 (fractal scaling)^17^. The value of the exponent in Eq. (4) obtained in the meta-analysis is consistent with values obtained using these *α* and *β* values; for example, *γ* = −0.535 (the value for *in situ* temperatures) can be obtained if *α* = 0.822 and *β* = −1.5; *γ* = −0.63 (the value at 15 °C) can be obtained if *α* = 0.75 and *β* = −1.66 (Supplementary Fig. S3). The consistency of the relationship is an encouraging sign, and warrants consideration of the mechanisms underlying the relationship and its usefulness for further study.

### Comparison with vegetation models

The negative relationship between organism size and the mass-specific metabolic rate is well known^18^ and is represented phenomenologically in vegetation and carbon cycle models^10,11^. These models use different *r*_A_ values for construction (growth-rate-dependent) and maintenance (standing-mass-dependent) respiration rates of leaves, stems, and roots. Thus, the allometric vegetation growth relationships assumed in models (e.g., the accumulation of woody tissues in mature forests) can be expected to simulate the size dependence of apparent *r*_A_. Several models also consider shifts in nitrogen concentrations, which are expected to correlate with synthetic activity, and the associated respiration rates of plant organs. I examined the mass–*r*_A_ relationship in contemporary models using simulations from the Multi-scale Terrestrial Model Intercomparison Project (MsTMIP)^19^ (Supplementary Figs. S4 and S5).

**Figure 3:**
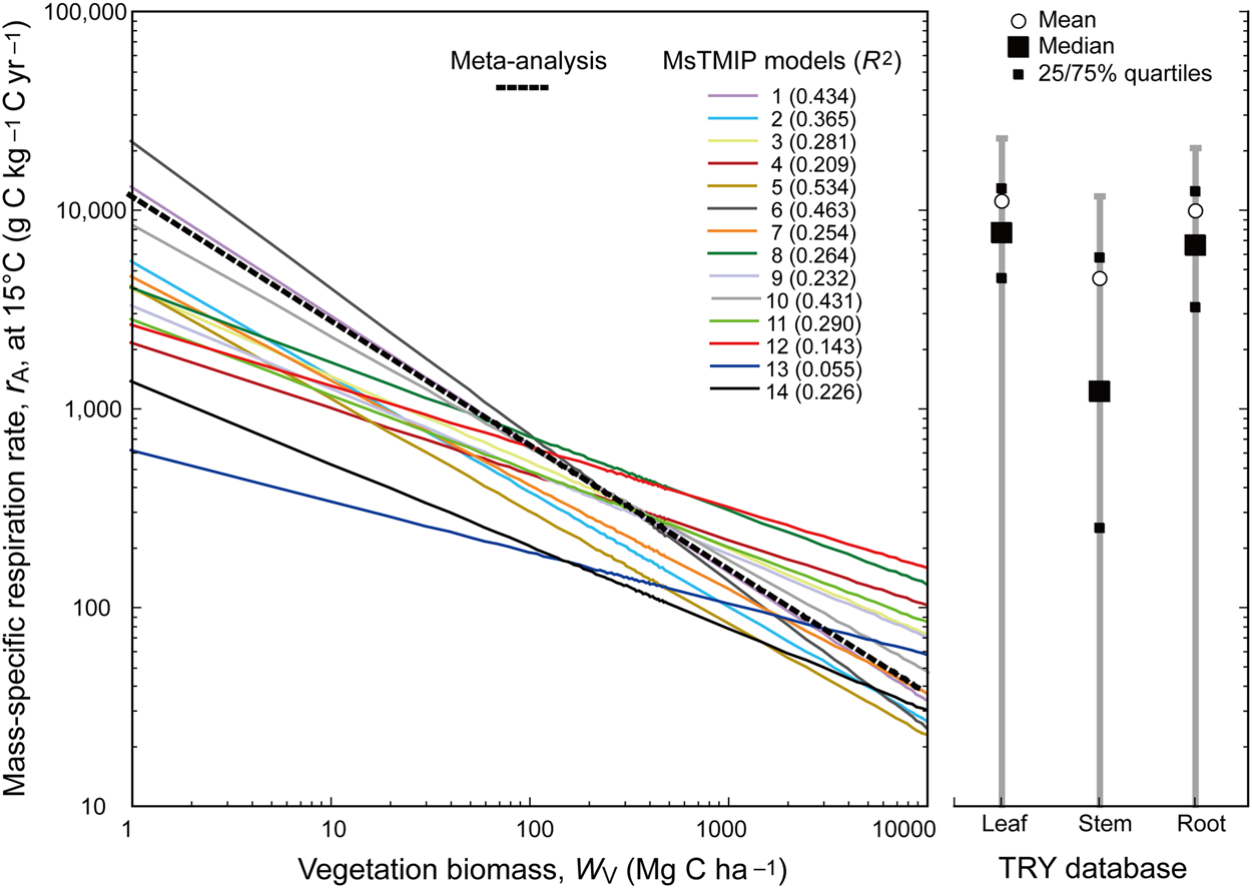
Relationship between vegetation biomass and mass-specific respiration rate in models from the Multi-scale model Intercomparison Project (MsTMIP). The dashed line shows the relationship obtained from the meta-analysis (see Fig. 1b). The right-hand panel shows the ranges and distributions of *r*_A_ values obtained from the TRY database^29^ for leaves, stems, and roots. All *r*_A_ values have been corrected to a common temperature of 15 °C. See Supplementary Fig. S5 for a comparison using uncorrected *r*_A_ values and a list of MsTMIP model names.

Analyses of global data with 14 models revealed a negative power-law relationship between grid-cell biomass and *r*_A_ (derived from *R*_A_ and *W*_V_), with exponents ranging from −0.662 to −0.167 at 15 °C (Fig. 3). These values are mostly less negative than the value of exponent *γ* obtained from the meta-analysis, implying a weaker mass-dependence of *r*_A_ in the models. Moreover, correlations between biomass and *r*_A_ were also weaker (*R*^2^ was 0.055–0.534 in the models and 0.765 in the meta-analysis). The mass–*r*_A_ relationship differed greatly among individual models, leading to different estimates of plant respiration rate at grid to global scales. Therefore, there is a clear need for an empirical relationship that could be used to constraint model parameterizations.

### Estimation of global *R*_A_

**Figure 4:**
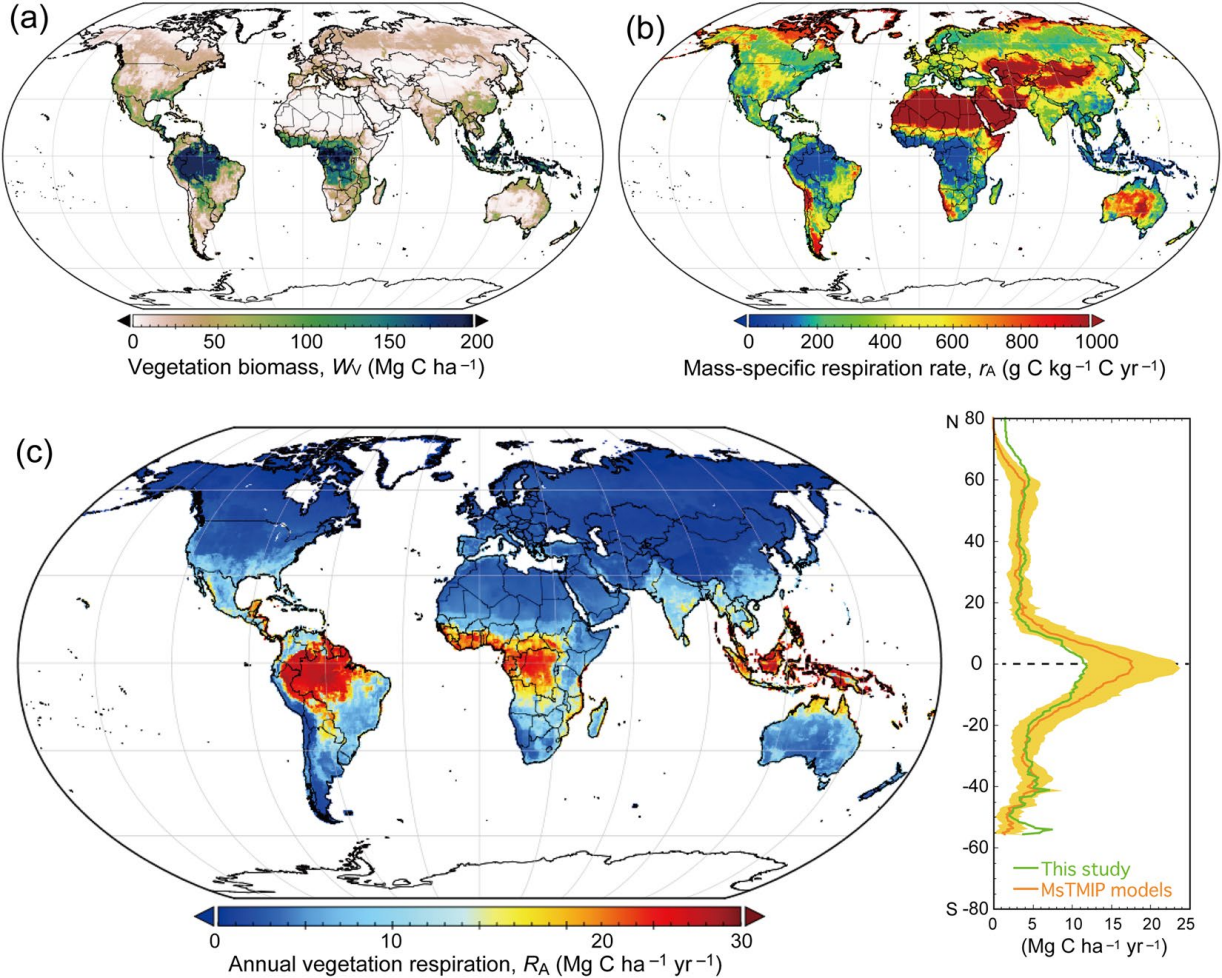
Global distribution of annual vegetation respiration rates. (**a**) Vegetation biomass, (**b**) mass-specific respiration rate estimated by using the relationship in Fig. 1a, and (**c**) vegetation respiration rate. To reduce biases caused by non-linearities in the scaling relationships, the calculation was made at 1-km resolution (as the highest available spatial resolution). The right-hand panel of (c) compares the latitudinal distribution of estimated respiration rates with those calculated from models in the Multi-scale Terrestrial model Intercomparison project (MsTMIP): lines show model means and the shaded area shows the standard deviation of the MsTMIP models.

To quantify the predictability of the mass–*r*_A_ relationship (Fig. 1a; *r*_A_ = 1194 · *W*_V_^−0.535^), I calculated *R*_A_ globally using a 1-km mesh map of plant biomass (*W*_V_). Annual global *R*_A_ was estimated to be 64.0 ± 12.0 Pg C yr^−1^ from 500 Pg C of plant biomass (Fig. 4), considering the uncertainty range of parameters. Assuming a typical value of global terrestrial GPP (120 Pg C yr^−1^)^20^, global NPP and carbon-use efficiency were estimated as 56 Pg C yr^−1^ and 0.467, respectively; these values are quite consistent with previous studies^6^. Although the *R*_A_ map is derived solely from biomass data, the distribution of estimated *R*_A_, which ranges from <1 Mg C ha^−1^ yr^−1^ in dry and cold climates to >20 Mg C ha^−1^ yr^−1^ in tropical forests, appears reasonable. Because of the size dependence of *r*_A_, forests account for only 58.1% of global *R*_A_ despite contributing 75.3% of global plant biomass. The mean and range of estimated *R*_A_ is comparable to estimates from previous studies. For example, in MsTMIP model results, *R*_A_ averaged 73.5 ± 20.0 (range, 48.2 to 120.8) Pg C yr^−1^ in 1991–2010. The inclusion of an independent estimate of heterotrophic respiration (51 Pg C yr^−1^) based on soil chamber observation data^21^ gives a total ecosystem respiration rate (the sum of autotrophic and heterotrophic respiration) of 115 Pg C yr^−1^, which is comparable to previous observation-based estimates and global CO_2_ syntheses (103–120 Pg C yr^−1^)^20,22^. The spatial distribution of *R*_A_ is similar to that of photosynthetic productivity, which is closely correlated with *R*_A_ (Supplementary Fig. S6), as estimated by up-scaling of flux measurements and remote sensing data. The difference in estimated *R*_A_ between the present study and the MsTMIP models implies that the models may overestimate *R*_A_ in the tropics (Fig. 4c), where high-biomass tropical rain forests predominate. Inadequate constraints on vegetation *r*_A_, perhaps caused by a failure to account for thermal acclimation in the models, may account for the difference.

### Biological implications

Variations in respiration or metabolic rate are likely to have profound biological implications that go beyond what us captured by simple gain–loss carbon accounting^1,23^. In the relationship presented in this study, *r*_A_ is determined by biomass and is independent of productivity. In reality, however, the lower *r*_A_ of developed ecosystems with high biomass is not only a consequence of senescence but also an adaptive strategy in resource-limited environments. In this study, I tested the power-law relationship and the behavior of its exponent in terms of plant metabolic scaling and demography, but my interpretation does not fully account for other factors such as temperature and nitrogen availability. Thermal acclimation of plant respiration may to some extent account for the large variations in plant respiration seen in the meta-analysis (e.g., the lower *r*_A_ of tropical forests than on boreal forests)^24,25^. See Supplementary Fig. S7 for a meta-analysis using a response function that includes thermal acclimation^26^. Notably, the regression line estimated using this response function has a larger exponent (i.e., a steeper biomass dependence of *r*_A_) but a lower coefficient of determination. In this regard, several chronosequence studies conducted at multiple nearby sites with different disturbance histories (e.g., elapsed time since the last fire) are useful to specify the scaling relationship, irrespective of temperature conditions. These studies have indicated that mass-specific respiration clearly decreases with ecosystem development (i.e., increasing total and mean individual biomass) (Supplementary Fig. S8). Nutrient limitation (e.g., nitrogen stoichiometry) may also affect this relationship through co-limitation and isometric scaling with plant nutrient content^27^. For example, plants subject to more severe nutrient limitation have a smaller biomass stock and need to invest more metabolic energy (associated with respiratory CO_2_) to extract and assimilate nutrients from the soil.

Despite the fact that *R*_A_ accounts for a large fraction of ecosystem CO_2_ emissions, it has not been adequately quantified to date. Total ecosystem respiration and soil respiration have been measured^5,28^, but their separation into emission components remains difficult. Most measurements of *R*_A_ in large plants, except for those using individual-tree chambers^14^ rely on indirect or destructive methods. Inadequate data quality and quantity have thus made examining *R*_A_ difficult. The recent compilation of various plant trait measurements into databases has facilitated global analyses of functional properties. Relevant data on plant leaf, stem, and root *r*_A_ in the public TRY database^29^ showed the range to be comparable to the estimate obtained in my meta-analysis (Fig. 3). The *r*_A_ values in the TRY database clearly differ among plant organs (from 4.5 ± 6.9 kg C kg^−1^ C yr^−1^ for stems to 11.1 ± 11.5 kg C kg^−1^ C yr^−1^ for leaves). Although this could enable a trait-based way to analyze plant properties globally^10^, mass-based information on plant respiration remains limited. The data cover only a fraction of plant diversity (1453 species for leaf respiration), and there are many fewer mass-based measurements than surface area-based measurements (e.g., for stem respiration, *n* = 26756 surface area-based measurements and 920 mass-based measurements). In addition to the expansion of trait-based databases, developments in remote sensing have provided more data that is relevant to terrestrial carbon budgets such as aboveground biomass^30^. Direct measurements of *R*_A_ from remote sensing platforms, however, remain out of reach.

## Conclusion

The present study provides an effective basis for reducing uncertainties in *R*_A_ values simulated in carbon cycle and Earth system models. As reported by previous global carbon cycle syntheses^22,31^, current evaluations of the global carbon cycle are still subject to considerable uncertainty. The present study may help constrain terrestrial ecosystem models, which have among the largest uncertainties. In practice, model parameters should be constrained or optimized so that the simulated *r*_A_ and *R*_A_ come close to the likely range expected from the empirical relationships. The non-linear relationship between vegetation biomass and *r*_A_ also highlights the necessity of high-resolution method to obtain accurate estimates of *R*_A_ in heterogeneous areas. The prospect of applying empirical constraints to dynamic vegetation models, which are being implemented in Earth system models, is especially promising. The *R*_A_ model described in this paper is certainly applicable to transitional states of vegetation associated with disturbance and climate change, and other mechanisms could be added to account for *R*_A_ responses to pollutants and extreme weather (e.g., droughts and heat waves) to fully explain variations in *r*_A_. Moreover, plant leaves have a second respiratory mechanism, photorespiration, which is regulated by different factors from the dark respiration that is the focus here. Integrating biological factors such as nitrogen dependence and thermal acclimation, in conjunction with empirical constraints as presented here, may further improve the parameterization of respiration.

## Methods

### Data collection for meta-analysis

Data used in the meta-analysis were obtained from two main sources – Internet searches on (1) Web of Science (Clarivate Analytics, Philadelphia, PA, USA) and (2) Google Scholar (Alphabet, Mountain View, CA, USA) – using keywords such as “autotrophic respiration”, “ecosystem”, “forest”, and “carbon cycle”. I collected original papers as much as possible and looked for data on ecosystem-scale autotrophic respiration, heterotrophic respiration, phytomass (plant biomass carbon stock), and soil organic carbon stock. I used observed annual values for respiration rates; to avoid extrapolation biases, daily to seasonal values were excluded. I also collected supplementary records from open-access datasets provided as part of several syntheses on the terrestrial carbon cycle^32–34^. Here again, I consulted original papers as much as possible to reduce data-extraction errors.

I then developed a database comprising records from the literature (Supplementary Table S1). Several sites reported multiple values derived by using different assumptions and correction methods; these were included in the analyses to assess the range of uncertainty caused by data handling. For each record, I collected site information for ancillary analyses: site latitude, land-cover type, annual mean temperature, annual precipitation, plant individual density, stand age (mostly for forests), basal area, canopy height, leaf area index, and so on. For ecosystem-scale carbon stock and respiration, units were standardized to Mg C ha^−1^ and Mg C ha^−1^ yr^−1^, respectively. Dry weight was converted to carbon weight by multiplying by a coefficient of 0.45; conversion from CO_2_ weight to carbon weight was done by multiplying by 12/44. For several studies, total autotrophic respiration rate was obtained by summing component fluxes from plant organs; data that lacked major components (e.g., only aboveground respiration) were therefore not used.

Most respiration measurements in these studies were conducted by the chamber method. Specific respiration rates of vegetation components (e.g., leaf, stem, and root) were measured with cuvettes and then scaled up to ecosystem scale. Few direct measurements of whole-ecosystem autotrophic and heterotrophic respiration have been conducted at ecosystem scale because of practical constraints. Note that ecosystem-scale fluxes measured by the eddy-covariance method quantify net ecosystem CO_2_ exchange only; photosynthetic assimilation and ecosystem respiration were then estimated from net fluxes by using appropriate separation methods such as non-linear regression.

Statistical analyses were conducted in SPSS Statistics v. 25 (IBM Inc., Armonk, NY, USA). To obtain 95% confidence intervals for the regression coefficients (e.g., scaling exponents in the form of power laws), bootstrapping was conducted 1000 times. The null model was based on the null hypothesis that vegetation respiration rate is independent of biomass (Supplementary Fig. S9).

### Temperature correction of plant respirationrates

In general, the temperature response function, *f*(*T*), is described as:

5 $$f(T,\,{\rm{in}}\,{\rm{K}})=\exp (-{E}_{{\rm{a}}}/{\rm{k}}\,T)$$

where *E*_a_ is the activation energy (0.6 eV for metabolic rate), and k is Boltzmann’s constant (8.62 × 10^−5^ eV k^−1^). The temperature response of plant (and microbial) respiration is often parameterized as an exponential function with a parameter Q_10_ (increase per 10 °C temperature rise) as:

6 $$f(T,\,{\rm{in}}\,^\circ {\rm{C}})={{{\rm{Q}}}_{10}}^{(T\mbox{--}{T}_{0})/10}$$

where *T*_0_ is the base temperature (for example, 15 °C) at which *f*(*T*) takes the value 1. In many models, this function is applied to maintenance respiration, whereas the construction respiration is assumed to be proportional to growth rate. Thus, as a result of changes in maintenance and growth components, the apparent *f*(*T*) can change through time and between places. When standardizing the respiration rates obtained under different temperature conditions, the data were divided by *f*(*T*) values to convert them into the value at the base temperature, for example:

7 $${R}_{{\rm{A}}(T=15)}={R}_{{\rm{A}}(T=25)}/{{{\rm{Q}}}_{10}}^{(25\mbox{--}15)/10}$$

In Fig. 1b, a Q_10_ value of 2.0 was used for this conversion. Moreover, as a result of thermal acclimation, Q_10_ varies seasonally and geographically. The relationship between temperature and foliar respiration Q_10_ has been summarized as follows^26^:

8 $${{\rm{Q}}}_{10(T)}=3.09-0.043\,T({\rm{in}}\,^\circ {\rm{C}})$$

### Terrestrial model simulation outputs

Global simulation outputs of *R*_A_ and *W*_V_ were derived from the MsTMIP^35^ dataset, available from https://daac.ornl.gov/cgi-bin/dsviewer.pl?ds_id=1225. This study uses outputs of 14 models, which provide data on autotrophic respiration and plant biomass at a spatial resolution of 0.5° × 0.5° in latitude and longitude. For carbon cycle models (GTEC, LPJ-wsl, ORCHIDEE, SiBCASA, VEGAS2.1, and VISIT), the results of the MsTMIP SG3 experiment were used. The models were driven by time-series data on atmospheric CO_2_, climate, and land-use change. For carbon–nitrogen models (BIOME-BGC, CLASS-CTEM-N, CLM4, CLM4VIC, DLEM, ISAM, TEM6, and TRIPLEX-GHG), the results of the MsTMIP BG1 experiment were used. The models were driven by time-series data on atmospheric CO_2_, nitrogen deposition, climate, and land-use change. For each model and cell, values of *R*_A_ and *W*_V_ were averaged for the period 1991–2010. When standardizing temperature at 15 °C, grid temperature was obtained from CRU TS3.2^36^.

### Plant trait TRY database

Values of *r*_A_ for different plant organs were downloaded from the TRY database^29^ (https://www.try-db.org/TryWeb/Home.php; accessed 30 July 2019). This study used the following open access datasets: “Leaf respiration rate in the dark per leaf dry mass (trait no. 41)” (*n* = 10,719), “Root respiration rate per root dry mass (trait no. 514)” (*n* = 1161), and “Stem respiration rate per stem dry mass (trait no. 519)” (*n* = 540). These data were obtained by many different researchers for various plant species under different observational conditions. For each organ, the average, standard deviation, median, and 25% and 75% quartiles were calculated.

### Global *R*_A_ estimation

The global value of *R*_A_ and its estimation range were obtained by applying the meta-analysis regression equation to the global map of vegetation biomass^37^ (Fig. 4a). The calculation was conducted at approximately 1 km (30″ in latitude and longitude) resolution. Global total *R*_A_ was estimated as 64.0 Pg C yr^−1^ by using the equation in Fig. 1a; a sensitivity analysis showed that it varies from 60.4 to 66.4 Pg C yr^−1^ with a ± 10% change in biomass at each grid. The range of estimation uncertainty was obtained by perturbing coefficients in the regression equation: from the meta-analysis, standard deviations were 159.0 for the multiplier coefficient and 0.0358 for the exponent. Also, vegetation biomass was perturbed by ±10% standard deviation in each cell. Equation coefficients and biomass were randomly sampled 1000 times, and used independently for estimation of global *R*_A_. Finally, the average and standard deviation were calculated.

## Supplementary information

Supplementary Information.

Supplementary Table S1.

## Data Availability

The meta-analysis dataset used in this study is available from the Figshare repository (10.6084/m9.figshare.10252694.v1) and is attached as Supplementary Table S1.
