# Supplementary material for: Constraining size-dependence of vegetation respiration rates
Source: Sci Rep. 2020 Mar 9;10:4304. doi: 10.1038/s41598-020-61239-0 (PMC7062890; doi:10.1038/s41598-020-61239-0)
Supplement: Supplementary file 1 — Supplementary Information. [file 41598_2020_61239_MOESM1_ESM.pdf]

Constraining size-dependence of vegetation respiration rates

Akihiko Ito [1,2*]

[1] National Institute for Environmental Studies, 16-2 Onogawa, Tsukuba 305-8506, Japan.
[2] Japan Agency for Marine-Earth Science and Technology, 3173-25 Showa-machi, Yokohama 236-0001, Japan

*Corresponding author: email: itoh@nies.go.jp

- Supplementary method: Derivation of equation (3)
- Figure S1–S9
- List of studies used in the meta-analysis

Derivation of equation (3). The mass-specific respiration rate (r_A) is defined as vegetation respiration rate (R_A) divided by vegetation biomass (W_V):

$$r_A = \frac{R_A}{W_V}. \tag{S1}$$

W_V, density (N), and individual mean biomass (W_I) are simply related as $W_V = N \cdot W_I$. Equation (2) can then be expressed as:

$$W_I = b \cdot \left(\frac{W_V}{W_I}\right)^{\beta} \quad \text{then} \quad W_I = b' \cdot \sqrt[1+\beta]{W_V^{\beta}} \tag{S2}$$

where b and b' are coefficients. R_A and individual respiration (r_I) are also simply related as $R_A = N \cdot r_I$, and using the metabolic scaling relationship (equation [1])), equation (S1) can be expressed as:

$$r_A = \frac{N \cdot r_I}{W_V} = a \cdot \frac{W_V}{W_I}\frac{W_I^{\alpha}}{W_V} = a \cdot W_I^{\alpha-1} \tag{S3}$$

Combining equations (S2) and (S3) gives the following relationship between r_A and W_V is obtained as follows:

$$r_A = c \cdot \left(\sqrt[1+\beta]{W_V^{\beta}}\right)^{\alpha-1} \tag{S4}$$

Equation (S4) is identical to equation (3).

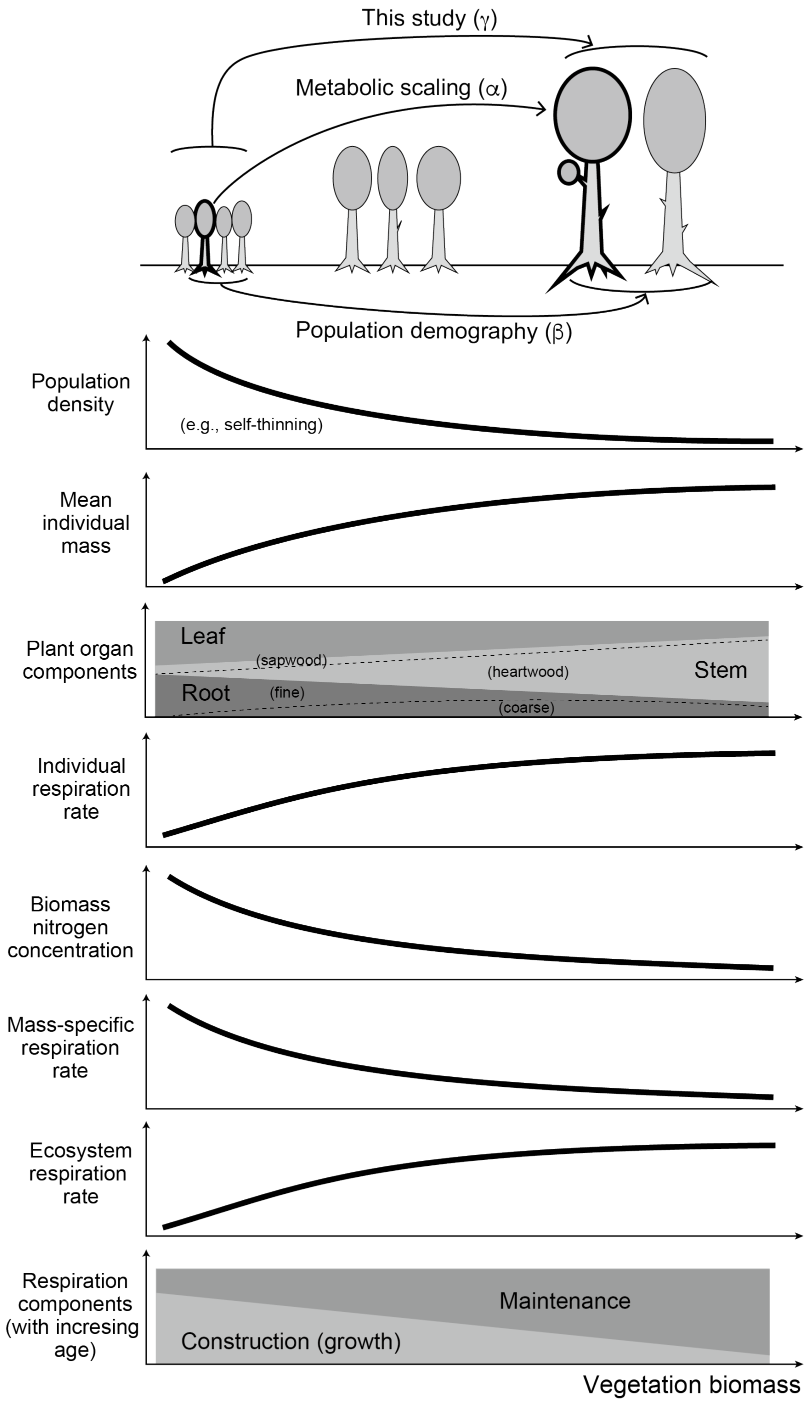

Fig. S1. Conceptual diagram of vegetation-level scaling with biomass. Changes in population density, mean individual biomass, individual respiration rate, biomass nitrogen concentration, mass-specific respiration rate, and total ecosystem respiration rate, as well as the composition of growth and maintenance respiration and plant organ components, are shown as functions of vegetation biomass.

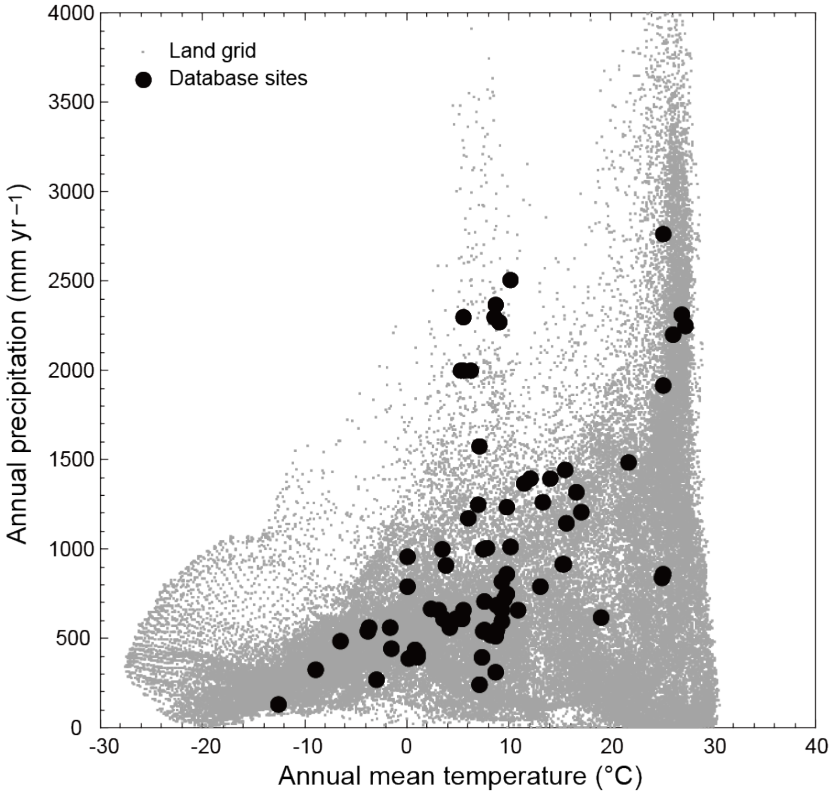

Fig. S2. Distribution of climate conditions in the terrestrial biosphere. Small gray dots show data from the CRU TS3.2 dataset [1] averaged over years 1981–2010 (30-year means) for all land grid points, and large black dots come from the observation dataset used in this study. Blank areas in the observation dataset represent warm and dry and cold and dry regions mostly occupied by barren polar and sandy deserts.

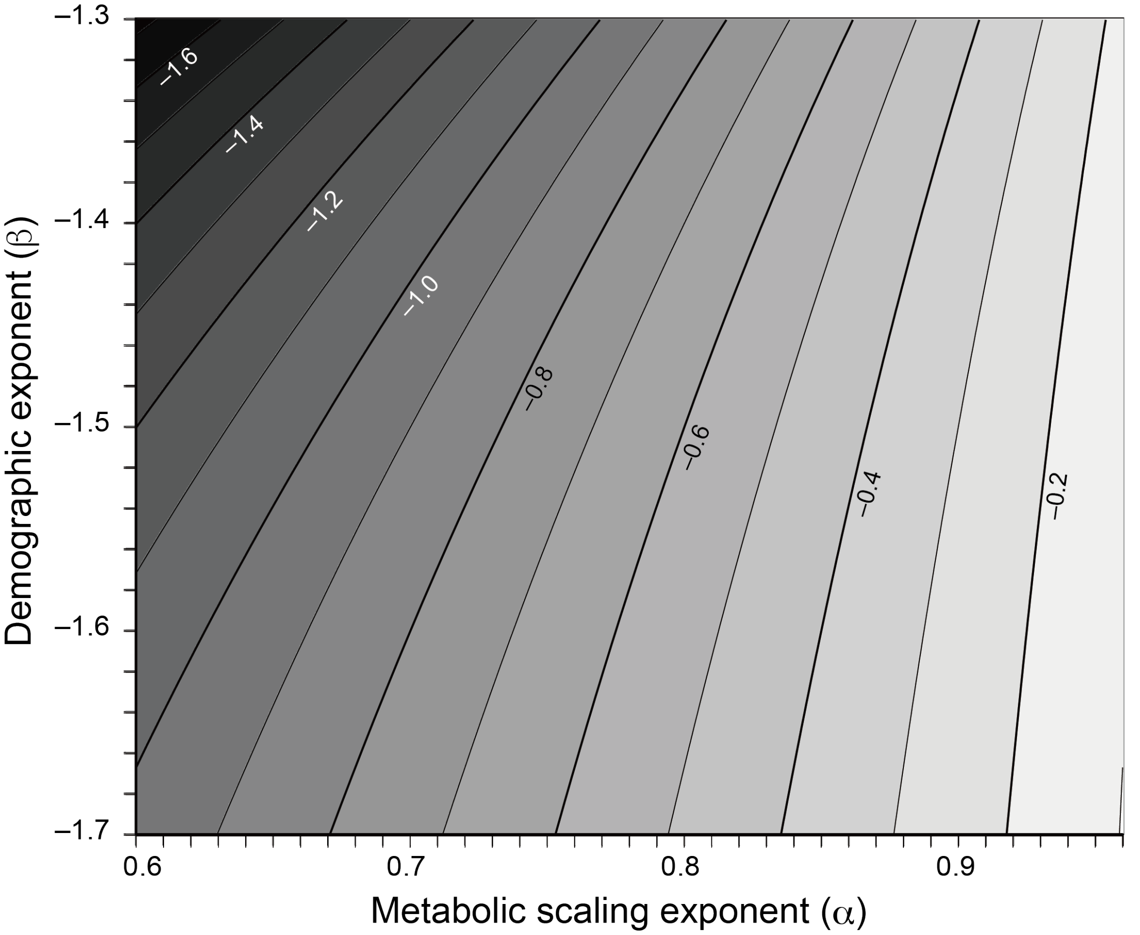

Fig. S3. Surface plot of the exponent (γ) of the mass-specific respiration rate equation as a function of the metabolic scaling exponent (α) and vegetation demographic exponent (β).

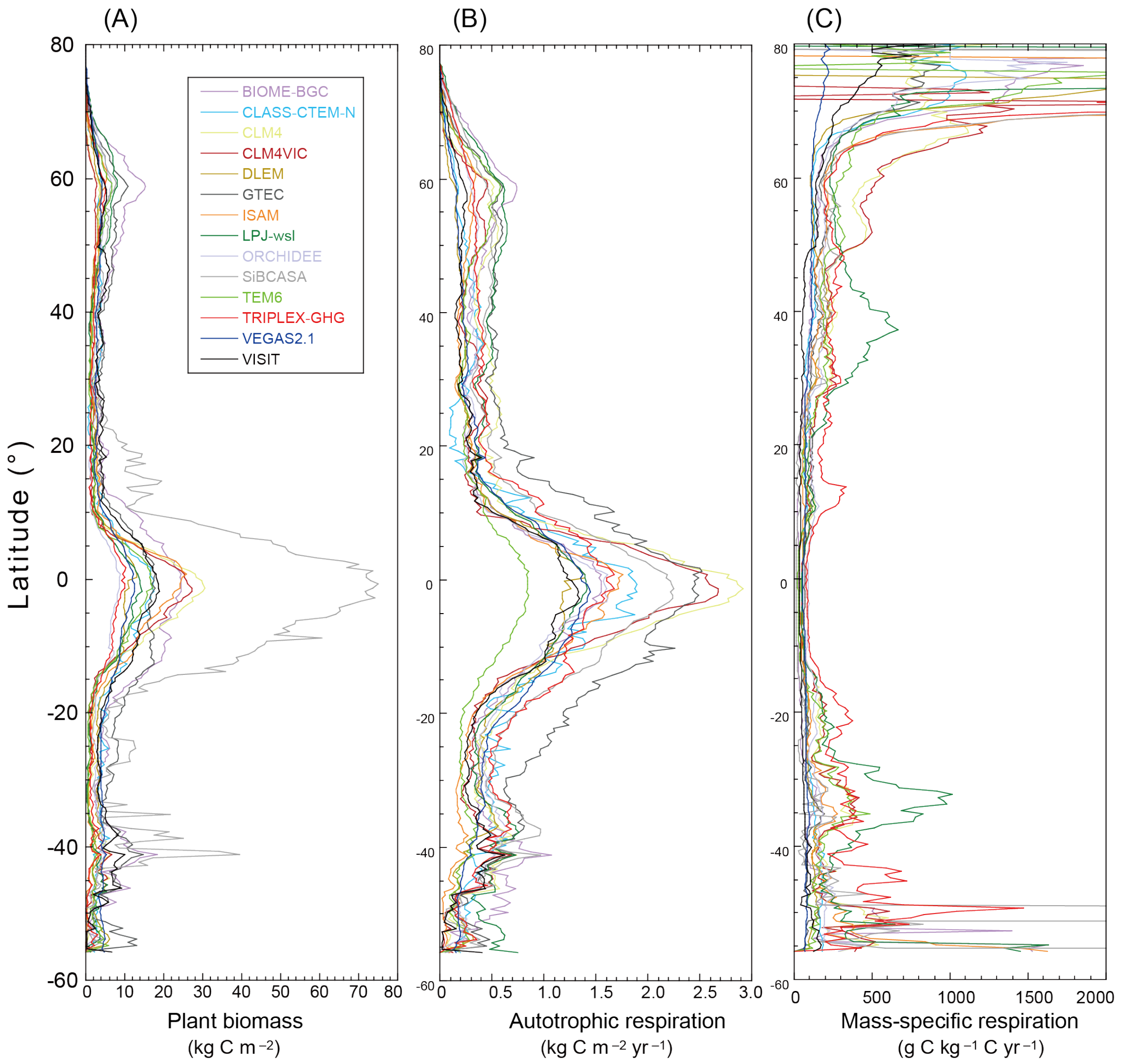

Fig. S4. Comparison of model outputs from the Multi-scale Terrestrial Model Intercomparison Project [2]. Panels show latitudinal distributions of (A) vegetation biomass, (B) annual autotrophic respiration, and (C) mass-specific respiration rate.

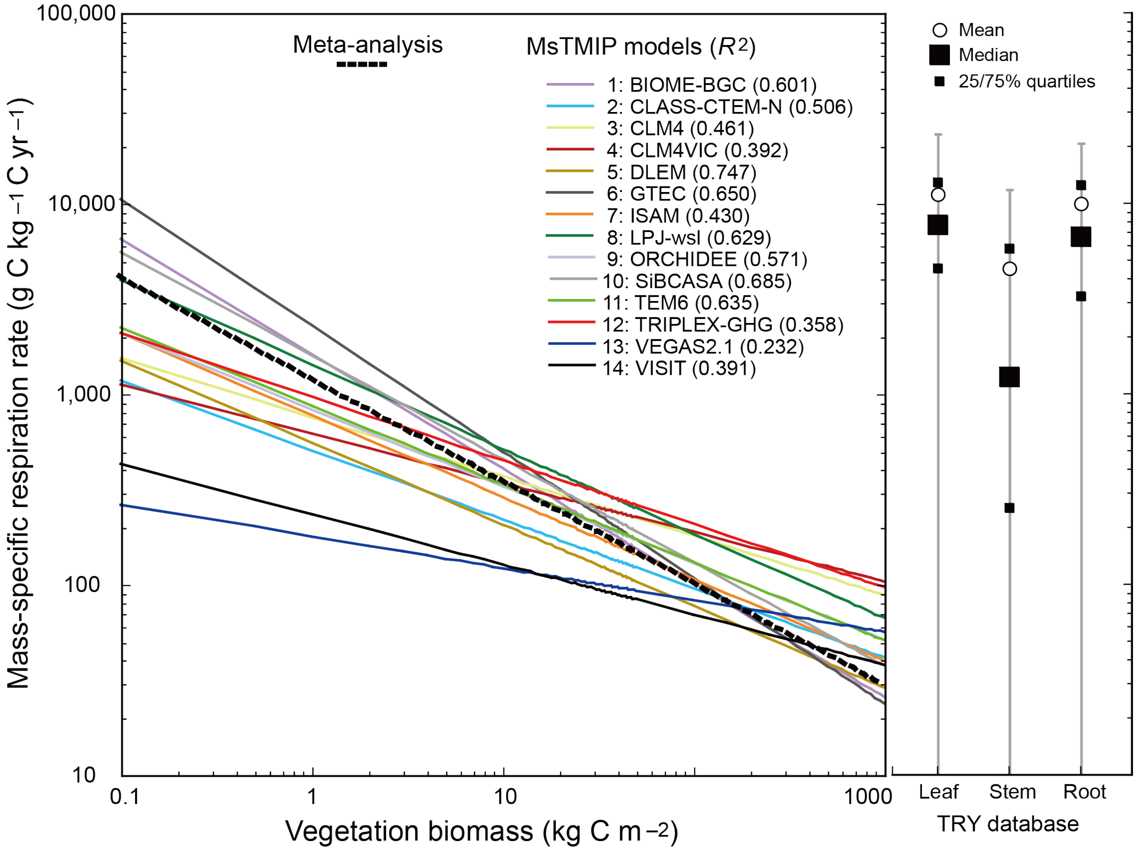

Fig. S5. Comparison of mass-specific respiration rate (r_A) at *in situ* temperature versus vegetation biomass among models from the Multi-scale Terrestrial Model Intercomparison Project (MsTMIP). The dashed line shows the relationship obtained from the meta-analysis (see Fig. 1a). The right-hand panel shows the ranged and distributions of organ r_A values obtained from the TRY database for leaves, stems, and roots.

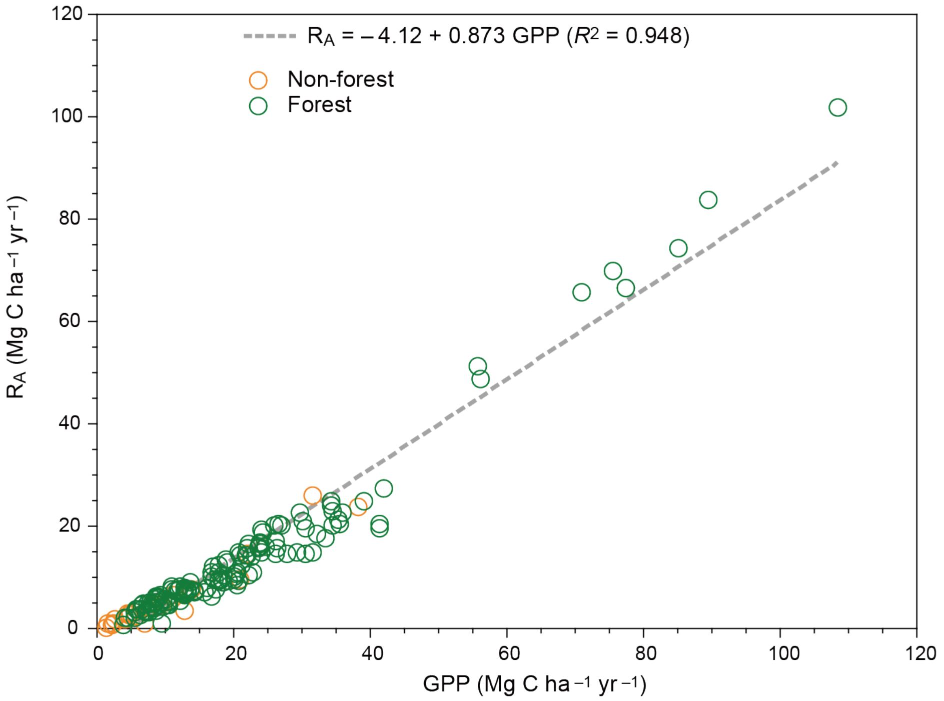

Fig. S6. Relationship between gross primary production (GPP) and ecosystem autotrophic respiration (R_A) in the meta-analysis dataset. The gray dashed line indicates the linear regression.

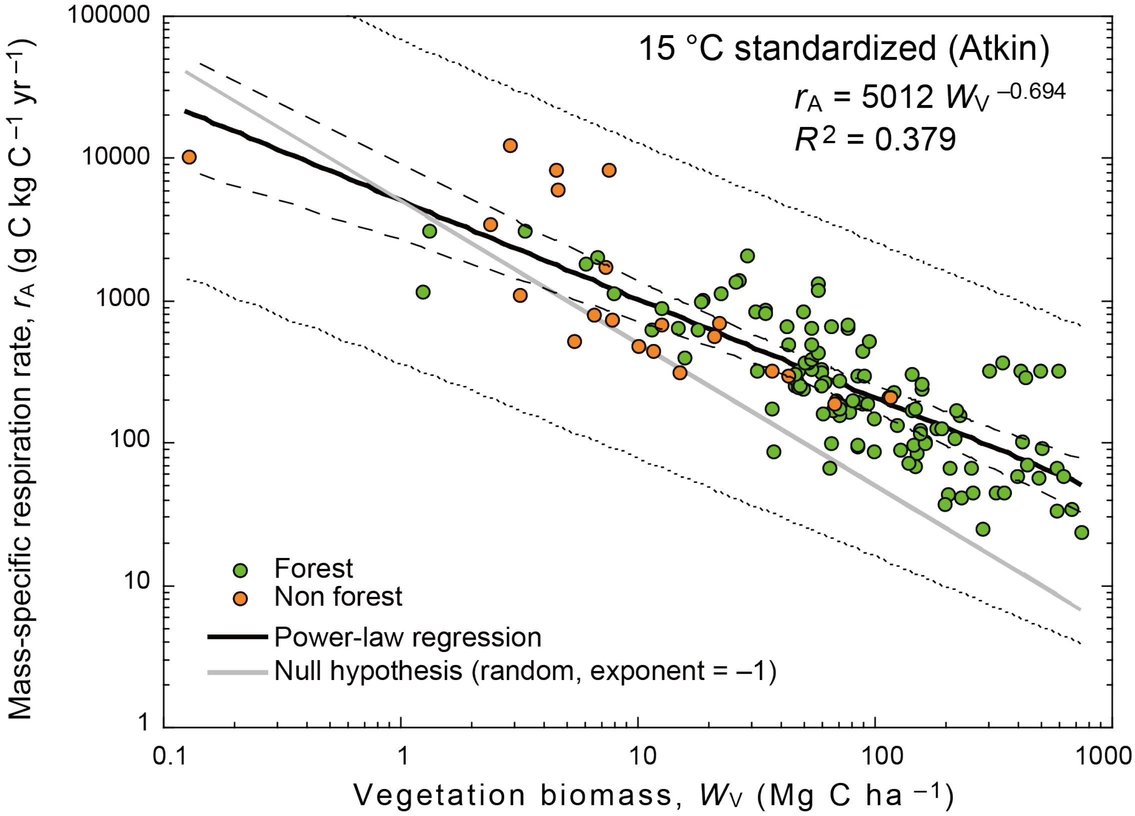

Fig. S7. Relationship between vegetation biomass and mass-specific respiration rate standardized to 15°C with a temperature response function including thermal acclimation [3]. The thick line shows a power-law regression (n = 144 and P < 0.001). Regression equations and correlation coefficients are shown in the figure. Dashed lines indicate 95% confidence intervals, and dotted lines show 95% prediction intervals. The gray line shows the slope (i.e., –1.0) of the null model, which assumes that vegetation respiration rate is independent of biomass (under this assumption, any significant trend would merely be due to autocorrelation). The standardization of r_A to 15 °C was conducted by using an exponential temperature dependence curve with Q_{10} including acclimation by Atkin and Tjoelker (2003) [3] (see text for the formulation).

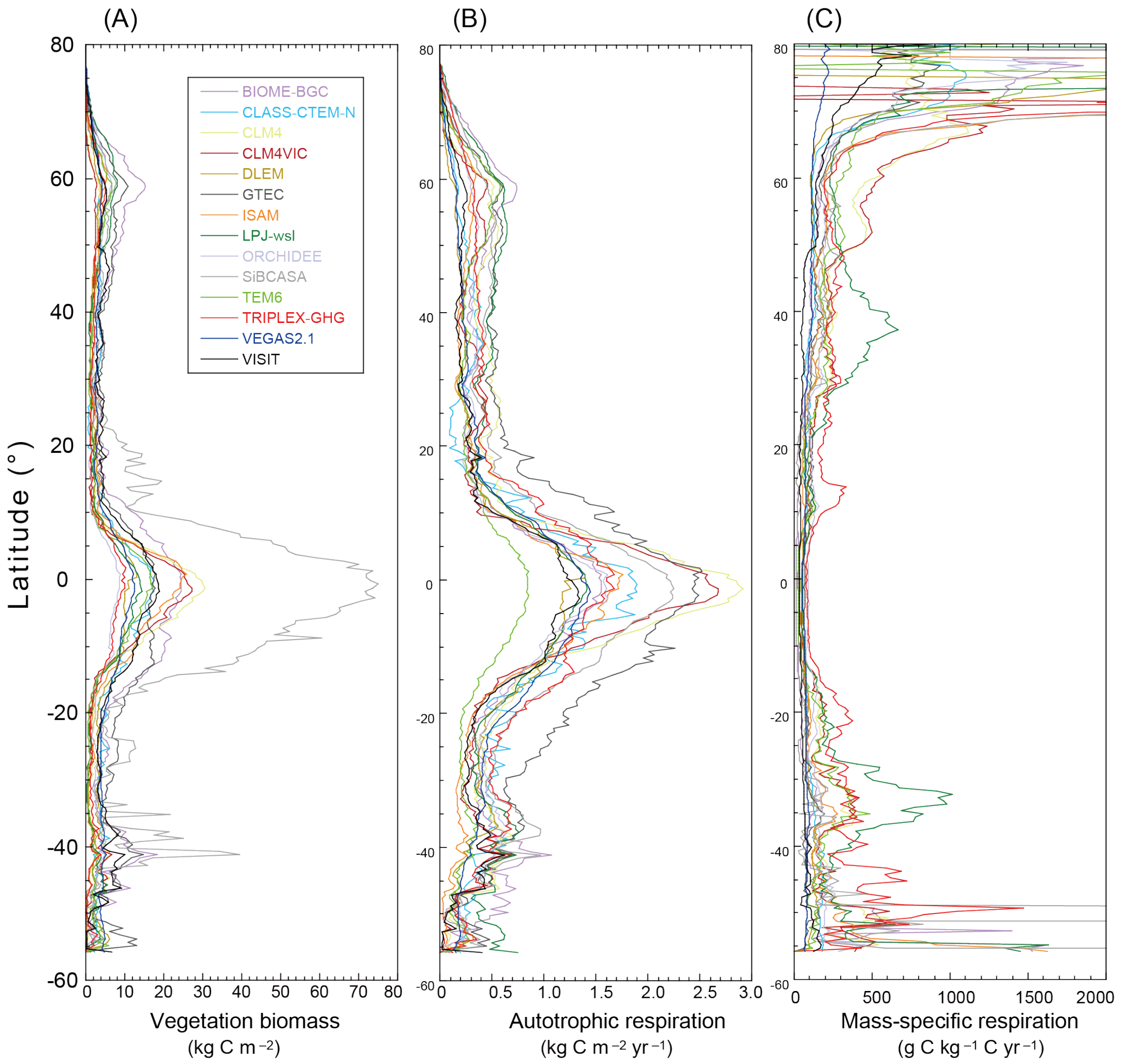

Fig. S8. Chronosequences of mass-specific respiration rates at five sites, Andrews, Cascade, Metolius, Turkey, and Fuji. Panels show relationships with (A) stand age, (B) vegetation biomass, and (C) stand density.

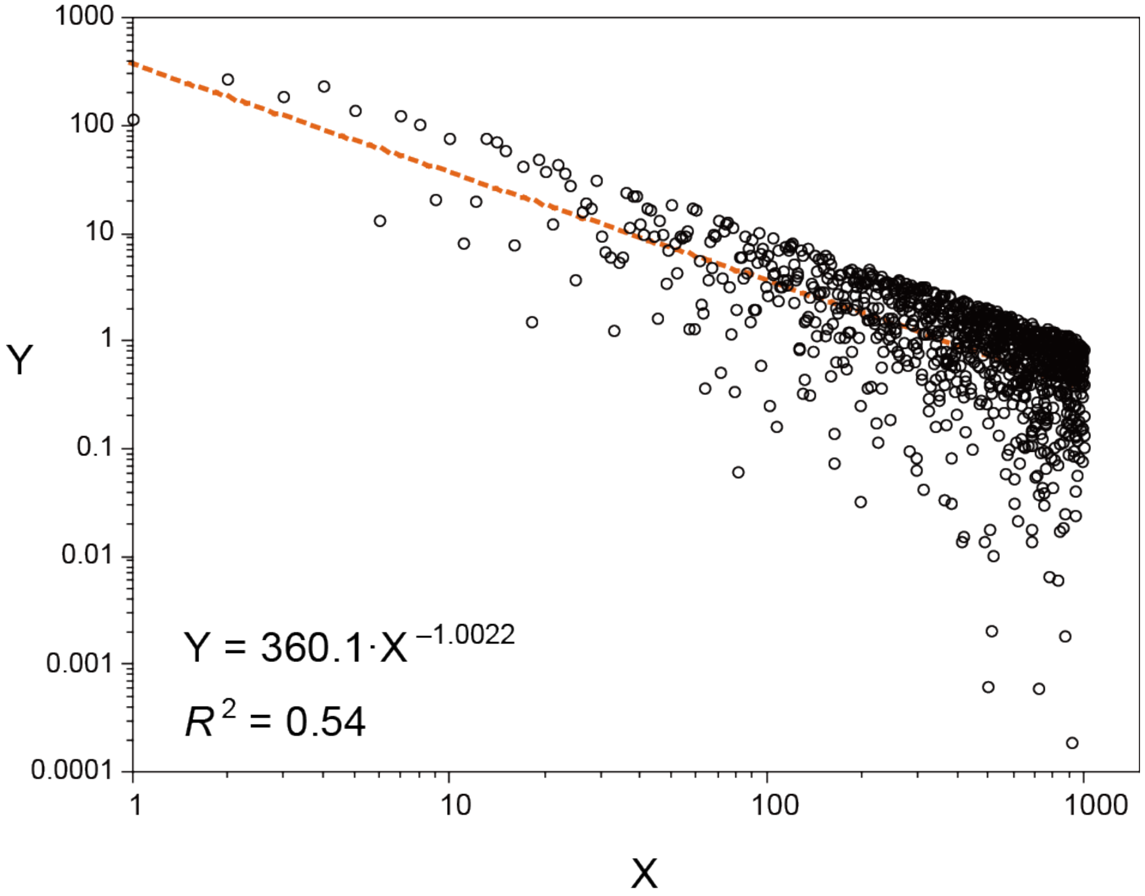

Fig. S9. Example of the null model output. Y values for each point were obtained by dividing random numbers by X-axis values (uniform with range 1–1000). The dotted orange line shows a power-law regression.

List of studies used in the meta-analysis

S1. A. Arneth, F. M. Kelliher, T. M. McSeveny, J. N. Byers, Net ecosystem productivity, net primary productivity and ecosystem carbon sequestration in a Pinus radiata plantation subject to soil water deficit. *Tree Physiology* **18**, 785–793 (1998).

S2. O. Bergeron et al., Comparison of carbon dioxide fluxes over three boreal black spruce forests in Canada. *Global Change Biol.* **13**, 89–107 (2007).

S3. W. D. Billings, Carbon balance of Alaskan tundra and taiga ecosystems: past, present and future. *Quaternary Sci. Rev.* **6**, 165–177 (1987).

S4. B. Bond-Lamberty, C. Wang, S. T. Gower, Contribution of root respiration to soil surface CO_2 flux in a boreal black spruce chronosequence. *Tree Physiology* **24**, 1387–1395 (2004).

S5. B. Bond-Lamberty, A. Thomson, Temperature-associated increases in the global soil respiration record. *Nature* **464**, 579–582 (2010).

S6. A. I. Breymeyer, B. Berg, S. T. Gower, D. Johnson, in Global Change: Effects on Coniferous Forests and Grasslands, A. I. Breymeyer, D. O. Hall, J. M. Melillo, G. I. Ågren, Eds. (Wiley, 1996), pp. 41–67.

S7. X. Chen, L. B. Hutley, D. Eamus, Carbon balance of a tropical savanna of northern Australia. *Oecologia* **137**, 405–416 (2003).

S8. H. Davi et al., Modelling carbon and water cycles in a beech forest. Part II.: Validation of the main processes from organ to stand scale. *Ecol. Model.* **185**, 387–405 (2005).

S9. E. H. DeLucia, D. J. Moore, R. J. Norby, Contrasting responses of forest ecosystems to rising atmospheric CO_2: Implications for the global C cycle. *Global Biogeochem. Cycles* **19**, doi:10.1029/2004GB002346 (2005).

S10. S. Dore et al., Carbon and water fluxes from ponderosa pine forests disturbed by wildfire and thinning. *Ecol. Appl.* **20**, 663–683 (2010).

S11. D. Epron, L. Farque, E. Lucot, P.-M. Badot, Soil CO_2 efflux in a beech forest: the contribution of root respiration. *Ann. Forest Science* **56**, 289–295 (1999).

S12. S. Etzold et al., The carbon balance of two contrasting mountain forest ecosystems in Switzerland: Similar annual trends, but seasonal differences. *Ecosystems* **14**, 1289–1309 (2011).

S13. M. Falk, K. T. Paw U, S. Wharton, M. Schroeder, Is soil respiration a major

contributor to the carbon budget within a Pacific Northwest old-growth forest? *Agr. For. Meteorol.* **135**, 269–283 (2005).

S14. M. Falk, S. Wharton, M. Schroeder, S. Ustin, K. T. Paw U, Flux partitioning in an old-growth forest: seasonal and interannual dynamics. *Tree Physiology* **28**, 509–520 (2008).

S15. J. Fang, G. Liu, B. Zhu, X. Wang, S. Liu, Carbon budgets of three temperate forest ecosystems in Dongling Mt., Beijing, China. *Science in China Ser. D* **50**, 92–101 (2007).

S16. K. Fenn, Y. Malhi, M. Morecroft, C. Lloyd, M. Thomas, The carbon cycle of a maritime ancient temperate broadleaved woodland at seasonal and annual scales. *Ecosystems* **18**, 1–15 (2015).

S17. M. L. Goulden et al., Patterns of NPP, GPP, respiration, and NEP during boreal forest succession. *Global Change Biol.* **17**, 855–871 (2011).

S18. S. T. Gower et al., Carbon distribution and aboveground net primary production in aspen, jack pine, and black spruce stands in Saskatchewan and Manitoba, Canada. *J. Geophys. Res.* **102**, 29029–29041 (1997).

S19. A. Granier et al., The carbon balance of a young Beech forest. *Func. Ecol.* **14**, 312–325 (2000).

S20. C. C. Grier, R. S. Logan, Old-growth *Pseudotsuga menziesii* communities of a western Oregon watershed: biomass distribution and production budgets. *Ecol. Monogr.* **47**, 373–400 (1977).

S21. T. J. Griffis et al., Seasonal variation and partitioning of ecosystem respiration in a southern boreal aspen forest. *Agr. For. Meteorol.* **125**, 207–223 (2004).

S22. J. G. Hamilton et al., Forest carbon balance under elevated CO_2. *Oecologia* **131**, 250–260 (2002).

S23. M. E. Harmon et al., Production, respiration, and overall carbon balance in an old-growth *Preudotsuga-Tsuga* forest ecosystem. *Ecosystems* **7**, 498–512 (2004).

S24. W. F. Harris, P. Sollins, P. Edwards, B. E. Dinger, H. H. Shugart, in Productivity of World Ecosystems. (1975), pp. 116–122.

S25. S. Hermle, M. B. Lavigne, P. Y. Bernier, O. Bergeron, D. Paré, Component respiration, ecosystem respiration and net primary production of a mature black spruce forest in northern Quebec. *Tree Physiology* **30**, 527–540 (2010).

S26. D. Hui, R. B. Jackson, Geographical and interannual variability in biomass

partitioning in grassland ecosystems: a synthesis of field data. *New Phytol.* **169**, 85–93 (2006).

S27. J. Irvine, B. E. Law, Contrasting soil respiration in young and old-growth ponderosa pine forests. *Global Change Biol.* **8**, 1183–1194 (2002).

S28. J. Järveoja, M. B. Nilsson, M. Gažovič, P. M. Crill, M. Peichl, Partitioning of the net CO_2 exchange using an automated chamber system reveals plant phenology as key control of production and respiration fluxes in a boreal peatland. *Global Change Biol.* **24**, 3436–3451 (2018).

S29. T. Kato et al., Temperature and biomass influences on interannual changes in CO_2 exchange in an alpine meadow on the Qinghai-Tibetan Plateau. *Global Change Biol.* **12**, 1285–1298 (2006).

S30. H. Keith, R. J. Raison, K. L. Jacobsen, Allocation of carbon in a mature eucalypt forest and some effects of soil phosphorus availability. *Plant Soil* **196**, 81–99 (1997).

S31. R. S. Kinerson, C. W. Ralston, C. G. Wells, Carbon cycling in a loblolly pine plantation. *Oecologia* **29**, 1–10 (1977).

S32. Y. Kominami et al., Biometric and eddy-covariance-based estimates of carbon balance for a warm-temperate mixed forest in Japan. *Agr. For. Meteorol.* **148**, 723–737 (2008).

S33. G. Kopittke, Universiteit van Amsterdam, Ph.D. Thesis (2013).

S34. P. M. Lafleur, T. R. Moore, N. T. Roulet, S. Frolking, Ecosystem respiration in a cool temperate bog depends on peat temperature but not water table. *Ecosystems* **8**, 619–629 (2005).

S35. B. E. Law, P. E. Thornton, J. Irvine, P. M. Anthoni, S. van Tuyl, Carbon storage and fluxes in ponderosa pine forests at different developmental stages. *Global Change Biol.* **7**, 755–777 (2001).

S36. A. Lindroth, L. Klemedtsson, A. Grelle, P. Weslien, O. Langvall, Measurement of net ecosystem exchange, productivity and respiration in three spruce forests in Sweden shows unexpectedly large soil carbon losses. *Biogeochemistry* **89**, 43–60 (2008).

S37. J. Lloyd et al., Seasonal and annual variations in the photosynthetic productivity and carbon balance of a central Siberian pine forest. *Tellus* **54B**, 590–610 (2002).

S38. Y. Luo, J. Reynolds, Y. Wang, D. Wolfe, A search for predictive understanding of

plant responses to elevated [CO_2]. *Global Change Biol.* **5**, 143–156 (1999).

S39. S. Luyssaert et al., CO_2 balance of boreal, temperate, and tropical forests derived from a global database. *Global Change Biol.* **13**, 2509–2537 (2007).

S40. C. A. Maier, T. J. Albaugh, H. L. Allen, P. M. Dougherty, Respiratory carbon use and carbon storage in mid-rotation loblolly pine (*Pinus taeda* L.) plantations: the effect of site resources on the stand carbon balance. *Global Change Biol.* **10**, 1335–1350 (2004).

S41. Y. Malhi, D. D. Baldocchi, P. G. Jarvis, The carbon balance of tropical, temperate, and boreal forests. *Plant, Cell, and Environ.* **22**, 715–740 (1999).

S42. Y. Malhi et al., Comprehensive assessment of carbon productivity, allocation and storage in three Amazonian forests. *Global Change Biol.* **15**, 1255–1274 (2009).

S43. T. R. Marthews et al., Simulating forest productivity along a neotropical elevational transect: temperature variation and carbon use efficiency. *Global Change Biol.* **18**, 2882–2898 (2012).

S44. A. D. McGuire et al., Interactions between carbon and nitrogen dynamics in estimating net primary productivity for potential vegetation in North America. *Global Biogeochem. Cycles* **6**, 101–124 (1992).

S45. P. C. Miller, R. Kendall, W. C. Oechel, Simulating carbon accumulation in northern ecosystems. *Simulation* **40**, 119–131 (1983).

S46. T. R. Moore, J. L. Bubier, S. E. Frolking, P. M. Lafleur, N. T. Roulet, Plant biomass and production and CO_2 exchange in an ombrotrophic bog. *J. Ecol.* **90**, 25–36 (2002).

S47. M. T. Nagy, I. A. Janssens, J. C. Yuste, A. Carrara, R. Ceulemans, Footprint-adjusted net ecosystem CO_2 exchange and carbon balance components of a temperate forest. *Agr. For. Meteorol.* **139**, 344–360 (2006).

S48. M. N. V. Navarro et al., Fruit development, not GPP, drives seasonal variation in NPP in a tropical palm plantation. *Tree Physiology* **28**, 1661–1674 (2008).

S49. A. Noormets et al., Response of carbon fluxes to drought in a coastal plain loblolly pine forest. *Global Change Biol.* **16**, 272–287 (2010).

S50. A. Noormets et al., The role of harvest residue in rotation cycle carbon balance in loblolly pine plantations. Respiration partitioning approach. *Global Change Biol.* **18**, 3186–3201 (2012).

S51. T. Ohtsuka, W. Mo, T. Satomura, M. Inatomi, H. Koizumi, Biometric based

carbon flux measurements and net ecosystem production (NEP) in a temperate deciduous broad-leaved forest beneath a flux tower. *Ecosystems* **10**, 324–334 (2007).

S52. T. Ohtsuka, N. Saigusa, H. Koizumi, On linking multiyear biometric measurements of tree growth with eddy covariance-based net ecosystem production. *Global Change Biol.* **15**, 1015–1024 (2009).

S53. M. Peichl, J. J. Brodeur, M. Khomik, M. A. Arain, Biometric and eddy-covariance based estimates of carbon fluxes in an age-sequence of temperate pine forests. *Agr. For. Meteorol.* **150**, 952–965 (2010).

S54. A. H. Rice et al., Carbon balance and vegetation dynamics in an old-growth Amazonian forest. *Ecol. Appl.* **14**, S55–S71 (2004).

S55. P. G. Risser et al., The true prairie ecosystem. (Hutchinson Ross, Stroudsburg, USA, 1981).

S56. M. G. Ryan, A simple method for estimating gross carbon budgets for vegetation in forest ecosystems. *Tree Physiology* **9**, 255–266 (1991).

S57. M. G. Ryan, R. M. Hubbard, S. Pongracic, R. J. Raison, R. E. McMurtrie, Foliage, fine-root, woody-tissue and stand respiration in Pinus radiata in relation to nitrogen status. *Tree Physiology* **16**, 333–343 (1996).

S58. M. G. Ryan, M. B. Lavigne, S. T. Gower, Annual carbon cost of autotrophic respiration in boreal forest ecosystems in relation to species and climate. *J. Geophys. Res.* **102**, 28871–28883 (1997).

S59. P. L. Sims, J. S. Singh, W. K. Lauenroth, The structure and function of ten western North American grasslands. *J. Ecol.* **66**, 251–285 (1978).

S60. E. W. Sulzman, J. B. Brant, R. D. Bowden, K. Lajtha, Contribution of aboveground litter, belowground litter, and rhizosphere respiration to total soil CO_2 efflux in an old growth coniferous forest. *Biogeochemistry* **73**, 231–256 (2005).

S61. Y. Tadaki, K. Hatiya, H. Miyauchi, Studies on the production structure of forest (XII) Primary productivity of *Abies veitchii* in the natural forests at Mt. Fuji. *J. Jpn For. Soc.* **49**, 421–428 (1967).

S62. K. Tan et al., Application of the ORCHIDEE global vegetation model to evaluate biomass and soil carbon stocks of Qinghai-Tibetan grasslands. *Global Biogeochem. Cycles* **24**, doi: 10.1029/2009GB003530 (2010).

S63. Z. Tan et al., Carbon balance of a primary tropical seasonal rain forest. *J. Geophys. Res*. **115**, doi:10.1029/2009JD012913 (2010).

S64. M. V. Thomas et al., Carbon dioxide fluxes over an ancient broadleaved deciduous woodland in southern England. *Biogeosciences* **8**, 1595–1613 (2011).

S65. M. S. Verlinden et al., Net ecosystem production and carbon balance of an SRC poplar plantation during its first rotation. *Biomass Bioenergy* **56**, 412–422 (2013).

S66. K. A. Vogt, C. C. Grier, C. E. Meier, R. L. Edmonds, Mycorrhizal role in net primary production and nutrient cycling in *Abies amabilis* ecosystems in western Washington. *Ecology* **63**, 370–380 (1982).

S67. K. Vogt, Carbon budgets of temperate forest ecosystems. *Tree Physiology* **9**, 69–86 (1991).

S68. R. H. Whittaker, G. M. Woodwell, Structure, production and diversity of the oak-pine forest at Brookhaven, New York. *J. Ecol.* **57**, 155–174 (1969).

S69. R. H. Whittaker, F. H. Bormann, G. E. Likens, T. G. Siccama, The Hubbard Brook ecosystem study: forest biomass and production. *Ecol. Monogr*. **44**, 233–252 (1974).

S70. M. Williams, P. A. Schwarz, B. E. Law, J. Irvine, M. R. Kurpius, An improved analysis of forest carbon dynamics using data assimilation. *Global Change Biol.* **11**, 89–105 (2005).

S71. G. M. Woodwell, D. B. Botkin, in Analysis of Temperate Forest Ecosystems, D. E. Reichle, Ed. (Springer-Verlag, New York, 1970).

S72. Y. Wu et al., Partitioning pattern of carbon flux in a *Kobresia* grassland on the Qinghai-Tibetan Plateau revealed by field ^{13}C pulse-labeling. *Global Change Biol.* **16**, 2322–2333 (2010).

S73. D. Zanotelli, L. Montagnani, G. Manca, M. Tagliavini, Net primary productivity, allocation pattern and carbon use efficiency in an apple orchard assessed by integrating eddy covariance, biometric and continuous soil chamber measurements. *Biogeosciences* **10**, 3089–3108 (2013).

References in the supplement

1 Harris, I., Jones, P. D., Osborn, T. J. & Lister, D. H. Updated high-resolution grids of monthly climatic observations – the CRU TS3.10 Dataset. *International Journal of Climatology* **34**, 623–642, doi:10.1002/joc.3711 (2014).

2 Huntzinger, D. N. *et al*. The North American Carbon Program Multi-scale Synthesis and Terrestrial Model Intercomparison Project: Part 1: Overview and experimental design. *Geoscientific Model Development* **6**, 2121–2133, doi:10.5194/gmd-6-2121-2013 (2013).

3 Atkin, O. K. & Tjoelker, M. G. Thermal acclimation and the dynamic response of plant respiration to temperature. *Trends in Ecology and Evolution* **8**, 343–351, doi:10.1016/S1360-1385(03)00136-5 (2003).
