# Supplementary material for: Constraining size-dependence of vegetation respiration rates
Source: Sci Rep. 2020 Mar 9;10:4304. doi: 10.1038/s41598-020-61239-0 (PMC7062890; doi:10.1038/s41598-020-61239-0)
Supplement: Supplementary file 2 — Supplementary Table S1. [file 41598_2020_61239_MOESM2_ESM.pdf]

Constraining size-dependence of vegetation respiration rates

Akihiko Ito [1,2*]

[1] National Institute for Environmental Studies, 16-2 Onogawa, Tsukuba 305-8506, Japan.

[2] Japan Agency for Marine-Earth Science and Technology, 3173-25 Showa-machi, Yokohama 236-0001, Japan

*Corresponding author: email: itoh@nies.go.jp

· Table S1

Supplementary Table S1. Dataset used in the meta-analysis. Measurement sites are listed from north to south.

Site name	Biome type	Plant biomass (Mg C ha^{-1})	Annual autotrophic respiration (Mg C ha^{-1} yr^{-1})	Mass-specific autotrophic respiration (g C kg-C^{-1} yr^{-1})	Latitude (° N)	Annual mean temperature (°C)	References (See reference list in Supplementary Materials)
Barrow, Alaska, USA	Tundra	4.6	0.8	173.5	71.3	−12.6	S3
Toolik Lake, Alaska, USA	Tundra	4.5	1.9	422.2	65.4	−9	S44
Toolik Lake, Alaska, USA	Tundra	7.5	3.2	426.7	65.4	−9	S44
Bonanza Creek Experimental Forest, Alaska	Boreal forest	90.0	3.3	36.7	64.8	−3	S44
Degerö Stormyr, Sweden	Boreal peatland, oligotrophic minerogenic more complex, northern Sweden, growing season	0.1	0.3	2588.0	64.2	2.3	S28
Flakaliden, Sweden	*Picea* boreal forest	43.1	5.9	137.5	64.1	3.1	S36
Knottasen, Sweden	*Picea* boreal forest	31.2	8.2	263.8	61.0	4.2	S36
Yenisei River, Russia	Pine boreal forest	53.5	3.8	71.3	60.8	−3.7	S37
Yenisey/Zotino, Russia	*Pinus* boreal forest	76.0	3.8	50.3	60.7	−6.5	S37, S39
Asa, Sweden	*Picea* boreal forest	84.5	7.8	91.9	57.1	7.5	S36
UCI-1989, BOREAS	Post-fire conifer forest	12.5	2.3	184.7	56.6	0.8	S17, S4
UCI-1964, BOREAS	Jack pine forest	18.7	3.9	209.4	55.9	0.8	S17, S4
UCI-1930, BOREAS	Black spruce forest	46.0	2.9	63.1	55.9	0.8	S17, S4
Thompson NSA (NOAS)	Boreal semiarid, deciduous, poplar forest	76.2	7.4	97.2	55.9	−1.6	S58, S39
Thompson NSA (NOJP)	Boreal semiarid, evergreen needle *Pinus* forest	65.5	6.5	99.4	55.9	−1.6	S58, S39
NSA-OBS, BOREAS	Old black spruce forest	57.2	8.3	145.1	55.9	−3.9	S58, S18
NSA-OJP, BOREAS	Old jack pine forest	29.0	6.5	224.6	55.9	−3.9	S58
NSA-OA, BOREAS	Old aspen forest	57.0	7.4	130.1	55.9	−3.9	S58
UCI-1981, BOREAS	Jack pine + aspen	18.5	3.7	201.8	55.9	0.8	S17, S4
UCI-1850, BOREAS	Black spruce forest	57.0	5.0	88.3	55.7	0.8	S17, S4
SSA-OBS, BOREAS	Old black spruce forest	49.2	7.9	159.6	55.6	0.1	S58
SSA-OJP, BOREAS	Old jack pine forest	34.6	5.4	154.8	55.6	0.1	S58
SSA-OA, BOREAS	Old aspen forest	93.3	9.1	97.3	55.6	0.1	S58
Schefferville, Quebec, Canada	Boreal woodland	22.0	2.9	130.0	54.7	0.0	S44
Prince Albert, Canada	Boreal forest	60.0	4.5	74.3	54.0	7.3	S41
Prince Albert SSA (SOBS), Canada	Boreal semiarid, evergreen needle *Picea* forest	53.7	4.5	83.1	53.9	1.0	S58, S39
Prince Albert SSA (SOJP), Canada	Boreal semiarid, evergreen needle *Pinus* forest	22.2	5.4	240.6	53.9	1.0	S58, S39
Prince Albert SSA (SOAS), Canada	Boreal semiarid, deciduous, poplar forest	155.8	8.1	51.7	53.6	0.5	S21, S39
Prince Albert SSA (SOAS), Canada	Boreal semiarid, deciduous, poplar forest	155.8	7.9	50.4	53.6	1.0	S58, S39
Oldebroek, control, Netherlands	*Calluna* heathland	10.0	3.0	300.0	52.4	10.1	S33
Oldebroek, young, Netherlands	*Calluna* heathland	7.3	7.8	1068.5	52.4	10.1	S33
Wytham Woods, UK	Temperate broadleaved woodland	116.0	14.7	127.0	51.8	9.7	S16, S64
Brasschaat, Belgium, Pinus	Temperate conifer forest	84.6	4.8	56.7	51.3	9.8	S47, S5
POPFULL, Belgium	Poplar forest	6.7	7.9	1177.9	51.1	9.5	S65
Black spruce, Quebec, Canada	*Picea-Pinus* mature forests	53.3	4.9	92.3	49.7		S2, S25
Beech, FR02, Hesse, France	Temperate forest	46.8	7.2	154.7	48.7	9.2	S19, S11, S8
Lägeren, Mountain forest, Switzerland	Forest containing *Fagus*, *Picea*, etc.	214.2	10.7	49.8	47.5	7.4	S12
Pacific silver fir, young, USA	*Abies amabilis*, 23yr, forest	36.9	1.2	32.5	47.3	5.5	S6, S66
Pacific silver fir, young, USA	*Abies amabilis*, 180yr, forest	98.5	5.5	55.9	47.3	5.5	S6, S66
Davos, Mountain forest, Switzerland	*Picea abies* forest	154.5	5.3	34.0	46.8	3.4	S12
Caldaro, Italy	Apple tree orchard	11.6	3.7	320.4	46.4	11.5	S73
Wind River, Cascade, Washington, USA	Douglas-fir forest	398.0	13.1	32.9	45.8	9.0	S13, S14, S23
Cedar Creek Natural History Area, Minnesota, USA	Temperate savanna	21.0	4.4	209.5	45.6	5.5	S44
Mer Bleue bog, Ottawa, Canada	Boreal bog	12.5	2.6	208.0	45.4	3.8	S46, S34
Cascade Head 3, Oregon, USA	*Tsuga* temperate forest	47.0	9.6	203.3	45.1	10.1	S35, S39
Cascade Head 4, Oregon, USA	*Tsuga* temperate forest	252.4	10.6	41.9	45.1	10.1	S35, S39
Cascade Head 5, Oregon, USA	*Tsuga* temperate forest	124.2	10.4	83.6	45.1	10.1	S35, S39
Cascade Head 1, Oregon, USA	*Tsuga* temperate forest	59.1	9.5	160.5	45.1	10.1	S35, S39
Cascade Head 2, Oregon, USA	*Tsuga* temperate forest	53.5	11.2	209.6	45.1	10.1	S35, S39
Cascade Head 6, Oregon, USA	*Tsuga* temperate forest	139.0	6.3	45.4	45.1	10.1	S35, S39
Cascade Head 7, Oregon, USA	*Tsuga* temperate forest	255.7	7.2	28.3	45.1	10.1	S35, S39
Cascade Head 8, Oregon, USA	*Tsuga* temperate forest	323.7	9.1	28.2	45.1	10.1	S35, S39

Cascade Head 11, Oregon, USA	*Tsuga* temperate forest	744.5	10.9	14.6	45.1	10.1	S35, S39
Cascade Head 9, Oregon, USA	*Tsuga* temperate forest	347.8	9.7	27.9	45.1	10.1	S35, S39
Cascade Head 10, Oregon, USA	*Tsuga* temperate forest	585.3	12.4	21.2	45.1	10.1	S35, S39
Cascade Head 12, Oregon, USA	*Tsuga* temperate forest	674.3	14.4	21.4	45.0	10.1	S35, S39
Ponderosa pine, mature, Oregon, USA	Ponderosa pine forest	127.3	5.7	44.9	44.5	8.1	S27, S35
Metolius 10, Oregon, USA	*Pinus* temperate forest	162.2	7.8	48.1	44.5	7.4	S35, S39
Metolius 9, Oregon, USA	*Pinus* temperate forest	188.9	11.1	58.5	44.5	7.4	S35, S39
Metolius 8, Oregon, USA	*Pinus* temperate forest	70.5	6.1	86.4	44.5	7.4	S35, S39
Metolius 6, Oregon, USA	*Pinus* temperate forest	92.1	8.2	89.1	44.5	7.4	S35, S39
Metolius 2, Oregon, USA	*Pinus* temperate forest	3.3	4.9	1461.1	44.5	7.4	S35, S39
Metolius 5, Oregon, USA	*Pinus* temperate forest	70.7	5.8	82.1	44.4	7.4	S35, S39
Metolius 1, Oregon, USA	*Pinus* temperate forest	17.9	5.3	294.6	44.4	7.4	S35, S39
Metolius 7, Oregon, USA	*Pinus* temperate forest	142.9	11.2	78.2	44.4	7.4	S35, S39
Ponderosa pine, Oregon, young	Ponderosa pine forest	14.6	4.5	304.0	44.4	7.5	S27, S35
Metolius 3, Oregon, USA	*Pinus* temperate forest	6.0	5.0	844.2	44.4	7.4	S35, S39
Metolius young, Oregon, USA	*Pinus* temperate forest	11.4	3.4	296.8	44.4	7.5	S70, S39
Metolius young, Oregon, USA	*Pinus* temperate forest	11.4	3.3	292.5	44.4	7.5	S70, S39
Metolius young, Oregon, USA	*Pinus* temperate forest	11.4	3.3	291.6	44.4	7.5	S70, S39
Metolius 4, Oregon, USA	*Pinus* temperate forest	47.1	5.4	115.5	44.4	7.4	S35, S39
Metolius 11, Oregon, USA	*Pinus* temperate forest	205.3	6.3	30.9	44.4	7.4	S35, S39
Metolius, Oregon, USA	*Pinus* temperate forest	161.5	7.5	46.5	44.4	7.4	S35, S39
Andrews 7, Oregon, USA	*Pseudotsuga* temperate forest	218.0	20.2	92.6	44.3	8.7	S35, S39
Andrews 8, Oregon, USA	*Pseudotsuga* temperate forest	504.0	25.1	49.8	44.3	8.7	S35, S39
Andrews 9, Oregon, USA	*Pseudotsuga* temperate forest	417.8	22.9	54.7	44.3	8.7	S35, S39
Andrews 3, Oregon, USA	*Pseudotsuga* temperate forest	26.7	20.2	758.2	44.3	8.7	S35, S39
Andrews 12, Oregon, USA	*Pseudotsuga* temperate forest	621.5	19.4	31.3	44.3	8.7	S35, S39
Andrews 1, Oregon, USA	*Pseudotsuga* temperate forest	34.2	16.0	467.4	44.3	8.7	S35, S39, S60
Andrews 6, Oregon, USA	*Pseudotsuga* temperate forest	182.4	12.4	68.2	44.3	8.7	S35, S39
Andrews 2, Oregon, USA	*Pseudotsuga* temperate forest	25.9	18.8	725.7	44.3	8.7	S35, S39
Andrews Experimental Forest Watershed 10, Oregon, USA	Temperate conifer forest	435.0	16.7	38.3	44.3	8.7	S44
Andrews 10, Oregon, USA	*Pseudotsuga* temperate forest	577.4	20.6	35.7	44.2	8.7	S35, S39
Andrews 4, Oregon, USA	*Pseudotsuga* temperate forest	119.8	14.5	121.2	44.2	8.7	S35, S39
Andrews 5, Oregon, USA	*Pseudotsuga* temperate forest	84.4	13.6	160.5	44.2	8.7	S35, S39
Andrews 11, Oregon, USA	*Pseudotsuga* temperate forest	488.8	15.1	31.0	44.2	8.7	S35, S39
Old douglas fir, Oregon, USA	*Pseudotsuga* menziesii, >450yr	432.1	66.7	154.3	44.0	8.5	S6
Douglas-fir, xeric north, Oregon, USA	Temperate forest, douglas-fir	407.7	70.0	171.7	44.0	8.5	S20
Douglas-fir, xeric south, Oregon, USA	Temperate forest, douglas-fir	593.3	101.9	171.7	44.0	8.5	S20
Douglas-fir, warm mesic, Oregon, USA	Temperate forest, douglas-fir	302.7	51.5	170.0	44.0	8.5	S20
Douglas-fir, mesic, Oregon, USA	Temperate forest, douglas-fir	493.0	83.8	170.0	44.0	8.5	S20
Douglas-fir, cool moist, Oregon, USA	Temperate forest, douglas-fir	343.7	65.8	191.4	44.0	8.5	S20
Hubbard Brook, Hew Hampshire, USA	Temperate deciduous forest	80.5	7.2	89.5	43.9	7.0	S69
Turkey Point, white pine, 6-yr, Ontario, Canada	White pine forest	7.9	4.3	546.8	42.7	7.8	S53
Turkey Point, white pine, 19-yr, Ontario, Canada	White pine forest	52.1	9.4	180.6	42.7	7.8	S53
Turkey Point, white pine, 34-yr, Ontario, Canada	White pine forest	68.2	6.7	97.9	42.7	7.8	S53
Turkey Point, white pine, 69-yr, Ontario, Canada	White pine forest	145.3	6.8	46.7	42.7	7.8	S53
Harvard Forest Hardwood Site, Massachusetts, USA	Temperate deciduous forest	155.0	7.6	49.0	42.5	6.0	S44
Harvard Forest, Massachusetts, USA	Temperate forest	148.0	10.2	68.9	42.5	6.0	S44
Curlew Valley, Utah, USA	Arid shrubland	5.4	1.3	231.5	41.1	7.1	S44
Brookheaven, Oak-pine forest, NY, USA	Temperate mixes forest	70.6	6.8	96.3	40.9	9.8	S71, S56
Brookhaven, Long Island, NY, USA	Oak-pine forest	49.4	7.3	146.7	40.9	9.8	S68
Central Plains Experimental Range, Colorado, USA	Short grassland	3.2	1.9	596.8	40.8	8.7	S44
Birch, Dongling Mt., China	Birch forest	47.9	3.6	74.5	40.0	3.6	S15
Oak, Dongling Mt., China	Oak forest	36.4	2.2	60.2	40.0	4.8	S15
Pine, Dongling Mt., China	Pine forest	50.5	6.9	137.2	40.0	5.4	S15
Haibei alpine meadow, China	Alpine meadow	2.4	1.2	513.4	37.6	−1.7	S29, S72, S62
Osage prairie, Oklahoma, USA	Temperate grassland	6.5	5.4	830.8	37.0	15.2	S44, S59

Oklahoma Osage grassland, USA	Grassland	7.8	5.8	748.4	37.0	15.2	S26, S55, S56
Takayama, Japan	Temperate forest	83.7	3.6	43.2	36.1	7.1	S51, S52
Loblolly pine, North Carolina, USA	*Pinus taeda*, 16yr	59.3	20.7	348.9	36.0	15.6	S6, S31
Haw River, 16yr, North Carolina, USA	*Pinus taeda*, North Carolina	70.6	20.7	292.8	36.0	15.6	S67
Loblolly pine, North Carolina, USA	Loblolly pine plantation	59.3	19.7	332.0	36.0	15.6	S31
Duke Forest FACE, ambient, USA	*Pinus taeda* forest	61.3	17.0	278.0	36.0	15.3	S22, S38, S9
Oak Ridge, Tennessee, USA	Temperate forest	98.1	7.8	79.7	36.0	14.0	S41
Liriodendron, 48yr, Tennessee, USA	*Liriodendron* temperate forest	84.6	14.4	169.7	35.9	13.3	S67
Mixed deciduous (Oak Ridge), Tennesseee, USA	Mixed deciduous forest	87.6	14.4	164.4	35.9	13.3	S24, S56
US-NC2, 15yr, USA	Loblolly pine forest	74.7	15.2	202.7	35.8	16.6	S49, S50
Loblolly pine, North Carolina, USA	Loblolly pine forest	77.6	14.8	190.7	35.8	16.6	S49
Coconino NF1, Arizona, USA	Mixed temperate forest	1.2	0.8	617.9	35.4	8.6	S10, S5
Coconino NF2, Arizona, USA	Mixed temperate forest	1.3	2.2	1689.4	35.4	8.8	S10, S5
Mt Fuji, Abies forest, 75 yr, Japan	Alpine conifer forest	142.2	16.3	114.4	35.4	5.5	S61
Mt Fuji, Abies forest, 55 yr, Japan	Alpine conifer forest	88.8	14.1	158.4	35.4	5.2	S61
Mt Fuji, Abies forest, 20 yr, Japan	Alpine conifer forest	42.3	11.2	265.1	35.4	6.2	S61
Centennial Forest, managed, Arizona, USA	Temperate conifer forest	46.1	6.6	144.1	35.1	9.3	S10, S5
Centennial Forest, managed, Arizona, USA	Temeprate conifer forest	31.5	5.9	185.5	35.1	9.3	S10, S5
Centennial Forest, natural, Arizona, USA	Temperate conifer forest	65.1	3.6	54.8	35.1	8.8	S10, S5
Centennial Forest, natural, Arizona, USA	Temperate conifer forest	65.7	6.3	95.4	35.1	9.1	S10, S5
SETRES, loblolly pine, control, North Carolina, USA	Loblolly pine plantation, control	15.7	7.6	482.8	34.9	17.0	S40
Yamashiro Experimental Forest, Japan	Red pine forest	63.9	4.5	69.8	34.8	15.5	S32
Chakia, India	Tropical deciduous forest	113.5	49.0	431.7	25.3	24.9	S44
Menglun, Xishuangbanna, China	Tropical seasonal forest	147.5	17.2	116.6	21.9	21.7	S62
Guanica State Forest, Puerto Rico	Xeromorphic savanna	43.0	26.0	604.7	17.9	25.0	S44
Caxiuana, Brazil	Tropical forest	231.0	21.4	92.6	−1.7	26.9	S42, S43
Manaus, Brazil	Tropical forest	281.0	14.8	52.7	−2.6	26.0	S41
Manaus, Brazil	Tropical forest	203.0	19.8	97.5	−2.6	27.3	S42, S43
Ducke Forest, Manaus, Brazil	Tropical forest	225.0	74.5	331.1	−2.8	26.0	S44
Tapajos, Brazil	Tropical forest	196.7	14.9	75.7	−2.9	25.0	S42, S54
Darwin, NT, Australia	Eucalypt open forest savanna	52.9	9.8	185.3	−12.5		S7
Cocoflux, Vanuatu	Palm plantation	36.3	24.0	660.8	−15.4	25.1	S48, S39
Nylsvley Nature Reserve, South Africa	Tropical savanna	15.0	6.7	443.3	−24.7	19.0	S44
BFG Plantation-C, Australia	*Pinus radiata* plantation	66.9	10.7	159.6	−35.35	13.0	S57, S5
Pierce's Creek Forest, control, Australia	*Pinus radiata* forest	74.4	10.7	143.6	−35.35		S22, S5
Unfertilized Piccadilly Circus, near Canberra, Australia	Dry sclerophyll eucalypt forest	144.9	12.2	84.2	−35.4		S30
Fertilized Piccadilly Circus, near Canberra, Australia	Dry sclerophyll eucalypt forest	138.7	11.2	80.5	−35.4		S30
Taita Experimental Station, North Island, New Zealand	Temperate evergreen forest	150.0	9.8	65.0	−41.2	12.0	S44
Balmoral Forest, New Zealand	*Pinus radiata* temperate forest	44.0	8.6	195.5	−52.9	10.8	S1
Arctic semi desert	Arctic semi desert	2.9	1.1	389.7		−12	S45
